# IL-23 drives uveitis by acting on a population of tissue-resident entheseal T cells

**DOI:** 10.1172/jci.insight.182616

**Published:** 2025-08-28

**Authors:** Robert Hedley, Amy Ward, Colin J. Chu, Sarah E. Coupland, Serafim Kiriakidis, Peter C. Taylor, Stephanie G. Dakin, ORBIT Research Consortium, Christopher D. Buckley, Jonathan Sherlock, Andrew D. Dick, David A. Copland

**Affiliations:** 1 Botnar Research Centre, and; 2 Sir William Dunn School of Pathology, University of Oxford, Oxford, United Kingdom.; 3 Academic Unit of Ophthalmology, Translational Health Sciences, University of Bristol, Bristol, United Kingdom.; 4 NIHR Biomedical Research Centre at Moorfields Eye Hospital and UCL Institute of Ophthalmology, London, United Kingdom.; 5 Liverpool Ocular Oncology Research Group, University of Liverpool, Liverpool, United Kingdom.; 6 ORBIT Research Consortium, Oxford, United Kingdom (detailed in Supplemental Acknowledgments).; 7 The Kennedy Institute of Rheumatology, University of Oxford, Oxford, United Kingdom.; 8 Janssen Research & Development, LLC, Spring House, Pennsylvania, USA.

**Keywords:** Immunology, Inflammation, Ophthalmology, Adaptive immunity, Cytokines, T cells

## Abstract

Recurrent acute anterior uveitis is a frequent extra-articular manifestation of the axial spondyloarthropathies (AxSpA): chronic inflammatory diseases affecting the spine, enthesis, peripheral joints, skin, and gastrointestinal tract. Pathology in AxSpA has been associated with local tissue-resident populations of IL-23 responsive lymphoid cells. Here we characterize a population of ocular T cell defined by CD3^+^CD4^–^CD8^–^CD69^+^γδTCR^+^IL-23R^+^ that reside within the anterior uvea as an ocular entheseal analogue of the mouse eye. Localized cytokine expression demonstrates that uveal IL-23R^+^ IL-17A–producing cells are both necessary and sufficient to drive uveitis in response to IL-23. This T cell population is also present in humans, occupying extravascular tissues of the anterior uveal compartment. Consistent with the concept of IL-23 as a unifying mediator in AxSpA, we present evidence that IL-23 can also act locally on tissue resident T cells in the anterior compartment of the eye at sites analogous to the enthesis to drive ocular inflammation.

## Abstract 2

Identification of tissue-resident IL-23-responsive T cells in the eye as orchestrators of ocular inflammation, offers common link to Axial Spondyloarthropathy related Uveitis.

## Introduction

Axial spondyloarthropathies (AxSpA) represent a group of chronic immune-mediated inflammatory diseases sharing clinical and molecular similarities, predominantly involving the axial skeleton but also peripheral joints and entheses (reactive arthritis [ReA]) (1–3). Frequently associated with these conditions are extra-articular manifestations including inflammation of the skin (psoriasis), gut (inflammatory bowel disease [IBD]), and eye (uveitis) (4, 5). Further evidence that these conditions are unified at cellular and mechanistic levels comes from the observations that, in patients with 1 disease, subclinical disease is often present at other anatomical sites. For example, AxSpA is strongly associated with subclinical gut inflammation (6) and IBD, and psoriasis is associated with subclinical entheseal inflammation (7).

Although the underlying pathogenic mechanisms of AxSpA are still not fully understood, strong genetic links implicate both human leukocyte antigen B*27 (HLA-B*27) allele, and the IL-23 pathway (8–17). The importance of IL-23 as a unifying factor for AxSpA is highlighted by single nucleotide polymorphisms (SNPs) in the IL-23 receptor (IL-23R), the IL-23 cytokine, and in genes involved in downstream signaling pathways and the IL-17 axis (18). The IL-23 pathway is recognized to play a prominent role at externally facing barrier surfaces, particularly the skin (19) and gut (20) but can also drive inflammation at internal sterile sites such as the joints (21). Resident myeloid cell populations found in both human entheses and barrier locations are recognized as the main source of local IL-23 production, capable of locally contributing to disease, although the in vivo triggers remain to be fully elucidated (22, 23). While barrier surfaces are characterized by the presence of an extensive microbiome, a fundamental feature of the joints is the presence of high biomechanical stress and tension.

IL-23R is constitutively expressed on various immune cell populations, including natural killer (NK) cells, innate lymphoid cells, γδ T cells, and mucosal-associated invariant T (MAIT) cells, all of which recognize structural elements via invariant T cell receptors or other recognition motifs. Engagement of IL-23R activates the intracellular Janus kinase/signal transducer and activator of transcription (JAK/STAT) signaling pathway with tyrosine-protein kinase (TYK2) and STAT3 being the dominant drivers for pathogenic Th17 cell cytokines (e.g., IL-17, IL-22, GM-CSF), which promote chronic tissue inflammation (24, 25). The ability of IL-23 to act rapidly at mucosal barrier tissues is partly due to the presence of resident type 17 cells, which express IL-23R (26). Tissue-resident IL-23–responsive CD3^+^CD4^–^CD8^–^ T cells are found at highly defined positions of the musculoskeletal (MSK) entheses in mice (21) and humans (27) as well as at other anchorage points associated with biomechanical stress including the aortic root and ciliary body (28, 29). Intriguingly, these tissue-resident cells are present even in the healthy state, which indicates their potential role in regulating barrier function, tissue repair, and homeostasis. The pattern of tissue localization of IL-23R–expressing cells can therefore determine how dysregulation of IL-23 biology can elicit inflammation at these precise anatomical sites.

Uveitis represents a heterogenous group of inflammatory disorders characterized by infiltration of leukocytes into the uveal tissues and intraocular cavities of the eye (30). Anterior uveitis, primarily affecting the iris and ciliary body, represents the most frequent type (approximately 80% of cases), resulting in vision loss secondary to cataracts, glaucoma, or macular oedema (31–33). Acute anterior uveitis (AAU) is the most severe form (34), presenting with acute onset of discomfort, eye redness, visual impairment, and cellular infiltration in the aqueous humor (AqH). AAU is classically described as the most common extra-articular manifestation in AxSpA, with one-third of patients developing intraocular inflammation (35). Patients with apparently isolated uveitis not only have a tendency for subclinical bowel inflammation (36), but also extensive subclinical enthesitis (37). HLA-B27–associated uveitis is often undiagnosed, and consequently, its association with AxSpA is overlooked (38, 39). HLA-B27 protein, present in up to 50% of patients with AAU, can misfold triggering the unfolded protein response, resulting in the production of IL-23 (40). In human studies, elevated serum levels of IL-23 are associated with an increased risk of AAU in patients with AxSpA (41) as well as other forms of uveitis, including Vogt-Koyanagi-Harada (VKH) and Behçet’s disease (BD) (42–44). Human genome-wide association studies (GWAS) demonstrate that in patients, SNPs in the IL-23R gene are associated with uveitis (45, 46). Irrespective of HLA-B27 positivity, AAU is also a feature of AxSpA-related diseases.

Collectively, these strong associations suggest common factors responsible for disease, with pathology orchestrated by resident populations of IL-23–responsive cells. Therapies neutralizing IL-23 in patients are effective in psoriasis, PsA, and IBD (47, 48). However, despite successes and insights, the biology and immunological etiology of human uveitis has remained enigmatic, in part due to the difficulty of obtaining healthy eye tissue and samples. We, therefore, sought evidence for a resident population of IL-23–responsive cells within the eye analogous to MSK entheses. Following the pivotal observation of γδTCR^+^ cells in the ciliary body in response to systemic IL-23 mini-circle (mc) expression (28), we show that IL-23 promotes intraocular inflammation in the mouse eye by acting on a previously uncharacterized population of CD3^+^CD4^–^CD8^–^CD69^+^γδTCR^+^IL-23R^+^ cells resident in the healthy anterior uvea, and that the expression of this cytokine alone, in the absence of other inflammatory signals, is sufficient to reproduce classical features of uveitis. Furthermore, data from postmortem human tissues demonstrates the extravascular location of resident CD3^+^γδTCR^+^ cells in the ciliary body and sclera, which secrete IL-17A upon activation. Our data support that the IL-23/IL-17 axis is an important therapeutic target in uveitis.

## Results

### Tissue-resident IL-23R^+^ γδ T cells are located in the murine anterior uvea.

**Figure 1:**
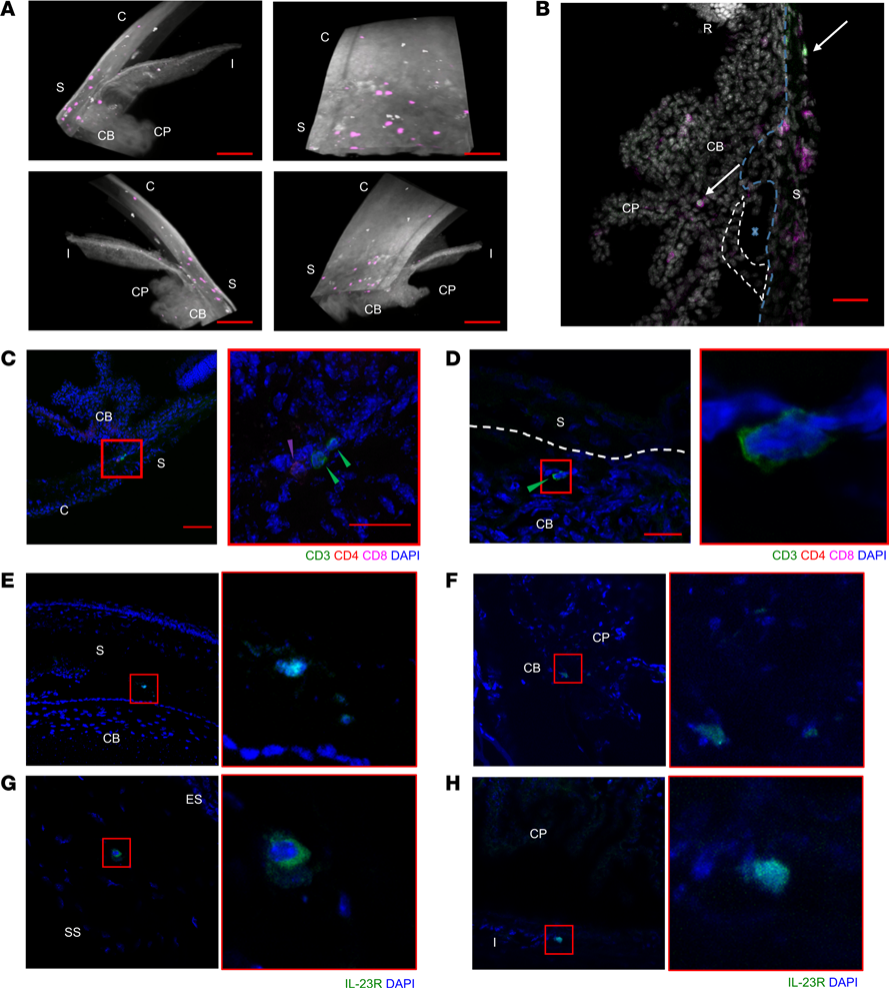
Tissue-resident CD3^+^IL-23R^+^ T cells are found in the mouse anterior uvea. Naive B6(Cg)-Tyrc-2J/J (albino) mice were perfused, and intact globes optically cleared, immunolabelled with CD3e and DAPI. (**A**) Lightsheet Z.1 acquired, immunofluorescent whole mount images (top left), rotated 90° (top right), rotated 180° (bottom left), and rotated 270° (bottom right). 3D rendered image shows expression of tissue autofluorescence (white) and CD3e (purple). Coexpression of CD3 and nuclei observed within different regions including the peripheral cornea, limbal sclera ,and ciliary body. Images captured at ×10 magnification. Scale bar: 100 μm. CP, ciliary process; CB, ciliary body; S, sclera; I, iris; C, cornea. (**B**) Immunofluorescence image of anterior tissue section from perfused albino mouse demonstrates presence of CD45^+^CD3^+^ T cells within extravascular tissues including the trabecular meshwork, ciliary body and sclera. Image captured at ×20 magnification. Dashed blue line, the inner limit of the sclera; dashed white line, trabecular meshwork; X, Schlemm’s canal; CP, ciliary process; CB, ciliary body; S, sclera; and R, retina. (**C**) CD4^+^ and CD8^+^ (single positive) cells are restricted to the juxtacanalicular tissue (JCT) region of the trabecular meshwork, proximal to the inner wall of Schlemm’s canal. Colored arrows indicating T cell subtypes; CD3^+^ (green), CD4 (red), and CD8^+^ (purple). (**D**) Sections also highlighted CD3^+^CD4^–^CD8^–^ (double-negative T cells) in the region of ciliary body proximal to the limbal sclera. (**E**–**H**) Sections taken from naive IL-23R–eGFP (+/–) reporter mice IL-23R^+^ cells are evident within the limbal sclera, specifically the transitional region of the sclera to ciliary body (**E**) and the transitional region from episclera (ES) to stromal sclera (SS) and not the vascular ES (**F**), the folding of the inner ciliary body (**G**), and posterior border layer of the iris (**H**). Nuclei (blue) and IL-23R–eGFP (green). Red square highlights region of tissue magnified in adjacent image (**C** and **E**–**H**).

Using light sheet fluorescence microscopy (LSFM), we assessed whether T cell populations are present within the anterior uvea and iridocorneal angle (ICA) tissue in adult albino [B6(Cg)-Tyrc-2J/J] mice. Perfused anterior segments were optically cleared (49), immunolabeled with anti-CD3 antibody and DAPI, before 3D cross-sectional images of the anterior chamber from different angles were captured. Low-magnification LSFM imaging of the anterior structure demonstrated clusters of CD3^+^ T cells located within different regions including the corneal limbus, sclera, and ciliary body (Figure 1A and Supplemental Figure 1; supplemental material available online with this article; https://doi.org/10.1172/jci.insight.182616DS1).

To determine their precise tissue location and phenotype, serial sections from perfused eyes were immunostained with additional surface markers, demonstrating that CD45^+^CD3^+^ cells reside within extravascular tissues including the trabecular meshwork (TM), ciliary body, and sclera (Figure 1B). CD4^+^CD8^–^ Th cells and CD4^–^CD8^+^ cytotoxic T cell (CD8^+^) populations are restricted to the juxtacanalicular tissue (JCT) region of the TM, proximal to the inner wall of Schlemm’s canal (Figure 1C). Interestingly, a population of CD3^+^CD4^–^CD8^–^ (double-negative T cells) was identified in the region of the ciliary body and ICA (Figure 1D), present in both the ciliary body and sclera parallel to the pars plana and pars plicata regions. Furthermore, using IL-23R–eGFP reporter mice, IL-23R^+^ cells are evident within the limbal sclera (Figure 1E) — specifically within the scleral stroma proper and not the vascular episclera (Figure 1F) — the ciliary body (Figure 1G) and posterior border of the iris (Figure 1H).

**Figure 2:**
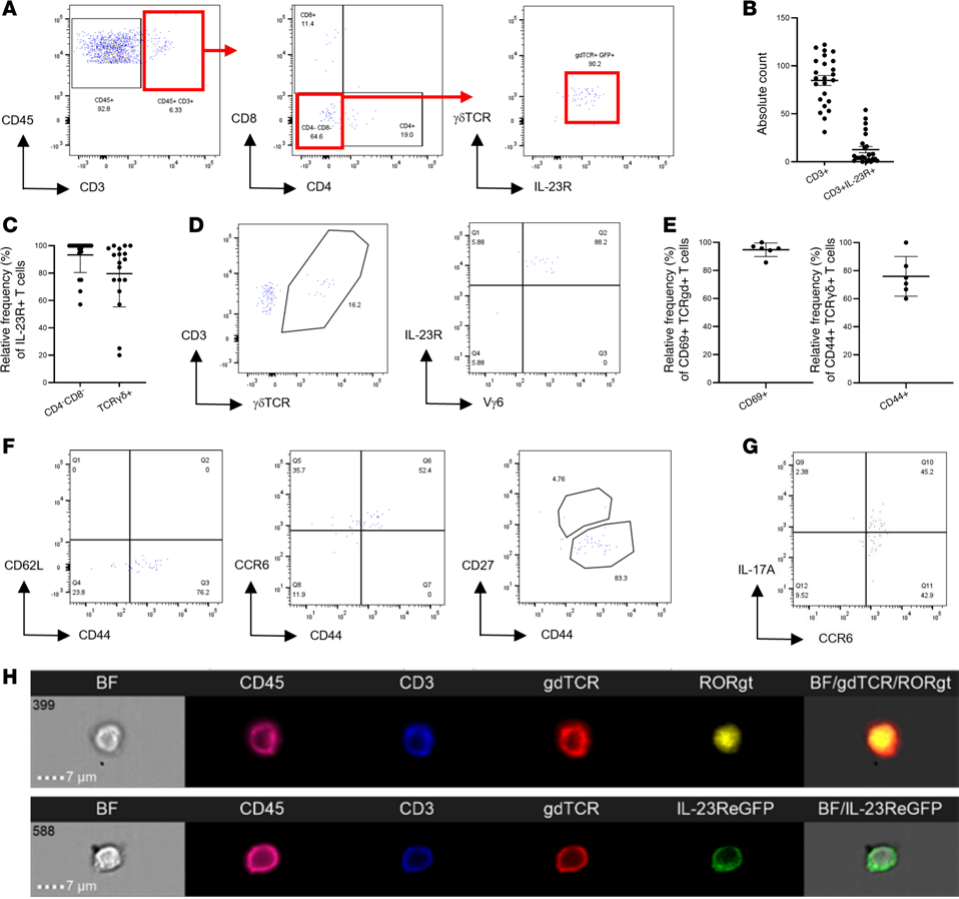
Naive anterior uvea contains CD3^+^γδTCR^+^IL-23R^+^ T cells with intrinsic pathogenic capacity. Anterior uvea tissue prepared from naive IL-23R–eGFP (+/–) reporter mice were analyzed by flow cytometry. (**A**) Representative flow plots showing gating strategy which identifies resident T cells as CD3^+^CD4^–^CD8^–^IL-23R^+^γδTCR^+^. (**B** and **C**) Graphs showing absolute counts of CD3^+^ and CD3^+^IL-23R^+^ populations isolated, and relative frequency of IL-23R^+^ cells expressing γδTCR^+^ (*n* = 25 AU samples). (**D**) Flow plots demonstrating CD3^+^IL-23R^+^γδTCR^+^ cells are Vγ6^+^ (no detectable expression of Vγ1 or Vγ4 chains), indicating an IL-17^+^ producing subset. (**E**) Relative frequency of IL-23R^+^ cells expressing CD69 and CD44 (*n* = 6 AU samples). (**F**) Resident IL-23R^+^γδTCR cells display a CD44^hi^CD62L^–^ (effector phenotype), expressing CCR6^+^ but not CD27. (**G**) Following ex vivo stimulation (PMA/ionomycin) of single-cell anterior uvea suspensions, intracellular cytokine staining demonstrates cells only secrete IL-17A. (**H**). Imagestream analysis of pooled naive anterior uvea suspensions (*n* = 4) confirms CD3^+^ IL-23R^+^ γδ^+^ surface phenotype, and intracellular expression of RORγt.

To define the phenotype and frequency of CD3^+^ populations, naive IL-23R–eGFP^+/–^ (heterozygous) mouse eyes (including nonpigmented albino background) were prepared for flow cytometry (Figure 2A and Supplemental Figure 2A). Enzymatic digestion of dissected anterior uveal tissue (including the limbal sclera, cornea, iris, and ciliary body) reveals that the IL-23R^+^ fraction represents 10%–15% of the total CD3^+^ T cell pool, and in absolute numbers, 50 cells can be routinely isolated from a single anterior uvea sample (Figure 2B). Furthermore, comparison of heterozygous and homozygous IL-23R–eGFP eyes reveals that IL-23R expression may represent a critical determinant in the residency/accumulation of this T cell population within a specific ocular location. In IL-23R–eGFP^+/+^ (homozygous) in which eGFP reporter sequences replace both IL-23R coding sequences rendering these mice functionally deficient in IL-23R, the total number and frequency of CD3^+^IL23R^+^ T cells is significantly reduced (Supplemental Figure 2B). Consistent with published observations of MSK entheseal tissues (21, 28), CD3^+^CD4^–^CD8^–^ γδ T cells, not αβ TCR^+^ T cells, constitute the majority of anterior compartment IL-23R^+^ T cells (Figure 2C and Supplemental Figure 2C). Typically, γδ T cell populations are classified according to the expression of the variable domain of the TCRγ (Vγ) chain (50). In the anterior compartment, all isolated γδ T cells were Vγ6^+^, with no detectable expression of Vγ1 or Vγ4 chains (Figure 2D and Supplemental 2D), suggesting an IL-17^+^ producing subset, as described for the MSK entheseal tissues, reproductive tract, lung, and skin (51, 52).

### Resident IL-23R^+^γδ^+^ T cells exhibit a preactivated effector phenotype.

Expression of IL-23R is a recognized hallmark of IL-17–producing cells, including Th17 cells (53–55), γδ T cells (56), and Type 3 innate lymphoid cells (ILC3) (57, 58). Furthermore, expression of C-C chemokine receptor 6 (CCR6) by T cells, including IL-17–producing γδ T cells (Tγδ17), is linked to increased IL-17A secretion (59). We therefore sought to characterize the IL-23R^+^γδ^+^ T cell phenotype to define their functional capacity.

Activated and functionally differentiated effector γδ T cells express CD69 and high levels of CD44 (51, 60). In naive mice, approximately 95% of anterior compartment-resident γδ T cells are CD69^+^ and CD44^hi^ (Figure 2E), and display a CD44^hi^CD62L^–^ effector phenotype (Figure 2F). Expression of CCR6 and CD27 permits functional discrimination between IL-17–producing and IFN-γ^–^ producing γδ T cell subsets (61). In the anterior compartment, the γδ T cells are CCR6^+^CD27^–^ (Figure 2F), and following ex vivo stimulation, secrete IL-17A but not IFN-γ (Figure 2G and Supplemental Figure 2E). Furthermore, ImageStream analysis confirmed the CD3^+^IL-23R^+^γδ^+^ surface phenotype and, importantly, highlights the intracellular expression/localization of retinoid orphan receptor γ t (RORγt) in cells isolated from the naive anterior compartment (Figure 2H).

These data demonstrate that healthy anterior uvea contains a resident T cell population, defined by surface marker expression, CD3^+^IL-23R^+^Vγ6^+^ γδ^+^CD69^+^CD44^hi^CCR6^+^. These cells are equipped with an intrinsic capacity to secrete IL-17A and, therefore, the potential to act as pathogenic effectors in the eye, comparable with the pathogenic cells described in psoriasis and in the MSK enthesis (21, 28, 62).

### IL-23 overexpression alone in the eye is sufficient to drive inflammation in vivo.

To evaluate IL-23 responsiveness of this resident T cell population in vivo, we engineered a ShH10 serotype adeno-associated virus (AAV) encoding a hyper–IL-23 cytokine (63) transgene to facilitate localized secretion of cytokine within the mouse eye (Supplemental Figure 3A). The ShH10 capsid serotype permits rapid transduction of the ciliary body nonpigmented epithelium and cells of the inner retina (Müller glia, ganglion cells, and astrocytes) when injected intravitreally into the mouse eye (64) (Supplemental Figure 3B).

**Figure 3:**
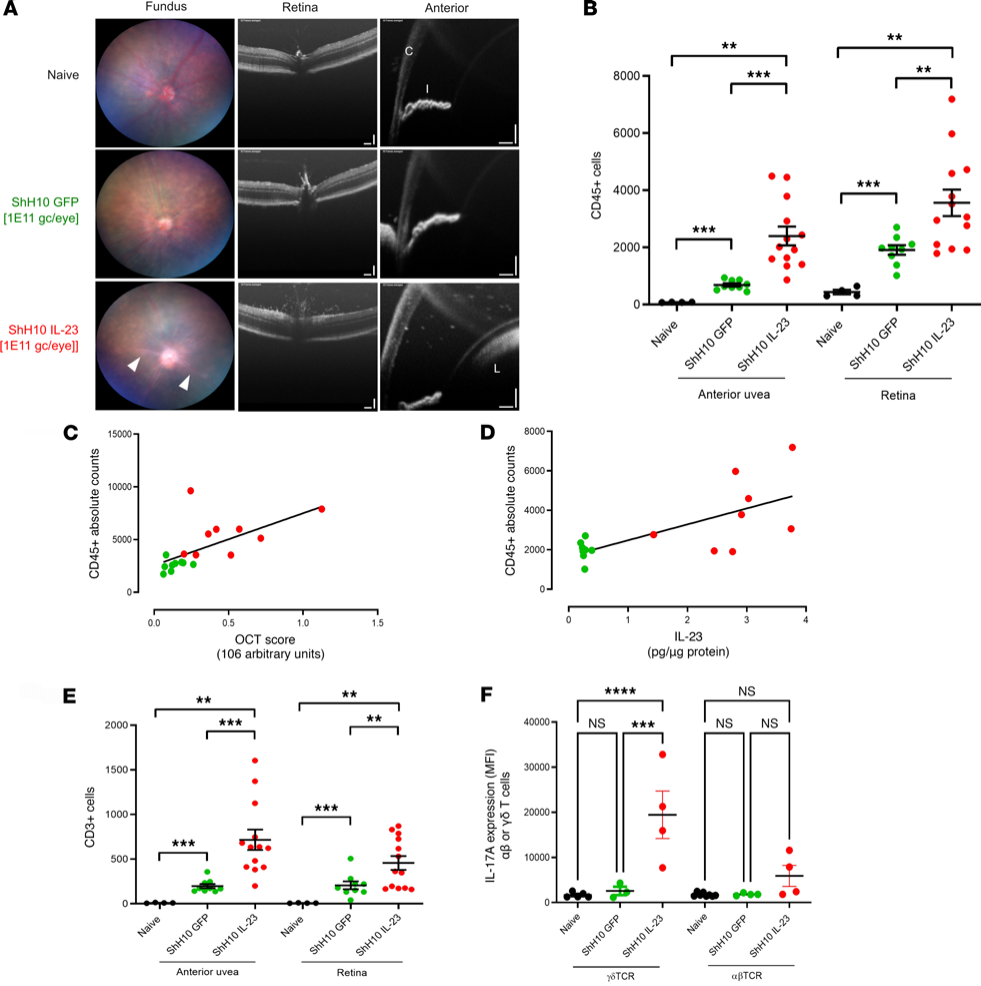
Ocular IL-23 expression drives increased CD45^+^ infiltration in the AU and retina. WT C57BL/6J mice received intravitreal injection of 1 × 10^11^ vector genomes of ShH10_IL23, with ShH10_GFP (control AAV) administered to the contralateral eye, and were clinically monitored until day 12. Enucleated eyes dissected and anterior uvea and retina samples prepared for flow cytometric immune phenotyping. (**A**) Representative fundus and OCT images of the retina and anterior uvea. White arrowheads indicate peri-vascular sheathing (fundus), posterior (vitreous), and anterior (aqueous) cell infiltrate in eyes receiving ShH10_IL23 virus. C, cornea; I, iris; L, lens. OCT scale bars: 100 mm. (**B**) Absolute CD45^+^ cell counts on day 12 from anterior uvea and retina samples from naive (*n* = 4), ShH10_GFP (*n* = 9) and ShH10_IL23 (*n* = 13). (**C** and **D**) Absolute counts correlated with OCT clinical disease score and IL-23 cytokine (protein) expression (*n* = 9/group). (**E**) Total live CD3^+^ counts in anterior uvea and retina on day 12. (**F**) Intracellular IL-17A expression (MFI, mean fluorescence intensity) following ex vivo restimulation from γδTCR^+^ or αβ TCR^+^ T cell subsets in response to ShH10_IL-23 (*n* = 4 eye from naive, ShH10_GFP or ShH10_IL-23 injected eyes). Statistical analysis was performed with 1-way ANOVA. Data are expressed as mean ± SEM. **P < 0.01, ***P < 0.001, **** *P* < 0.0001. Data shown are combined from 2 independent experiments (**B** and **E**) or from single representative experiment (**C**, **D**, and **F**).

Following intravitreal injection of 1 × 10^11^ vector genomes (vg)/eye of ShH10_IL-23 vector in C57BL/6J (WT) mice, posterior (retinal) inflammation, subtle optic disc swelling, retinal vascular changes, and cellular infiltration of the vitreous and aqueous cavities are manifest by day 12 (Figure 3A). In eyes receiving a control virus (ShH10_GFP; expressing a widely used reporter protein [GFP] and accepted to be noninflammatory) or vehicle (PBS) injection, no clinical inflammation or pathological changes are observed at the same time point. We performed immune profiling using flow cytometry to characterize the immune cell populations infiltrating both the anterior and posterior compartments. This demonstrates that IL-23 overexpression leads to a significant increase of CD45^+^ cells in each ocular compartment (Figure 3B), comprising both adaptive but also innate immune populations (Supplemental Figure 4). ShH10_GFP (control AAV) also results in subclinical accumulation of CD45^+^ cells compared with the naive eye, despite no obvious signs of inflammation, which reflects recognized AAV-mediated changes in the immune threshold of the tissue following intravitreal injection (65, 66).

In eyes receiving ShH10_IL23 virus, increased CD45^+^ counts correlate with higher clinical disease severity (Figure 3C) and expression levels of IL-23 protein detected by ELISA from tissue supernatants (Figure 3D). Phenotypic analysis of the CD45^+^ population shows that adaptive CD3^+^ cells (both CD4^+^ and CD8^+^) are the predominant cell type recruited to the eye in response to IL-23 expression (Figure 3E and Supplemental Figure 4). Ex vivo restimulation (PMA/ionomycin) of anterior uvea drives significantly higher expression levels of IL-17A from the γδ T cell subset compared with αβ TCR^+^ T cell subset in eyes receiving ShH10_IL-23 (Figure 3F). To evaluate the long-term outcome of IL-23 overexpression in the eye, we also monitored mice over an extended time course, until day 50 after intravitreal injection. Clinical imaging (fundus and OCT) demonstrates that eyes receiving the ShH10_IL-23 progress to develop a chronic and persistent inflammation, in contrast to control (ShH10_GFP) eyes that remain normal. FACS of combined AU and retina confirms disease severity, with a 10-fold increase in the total CD45^+^ and CD3^+^ absolute counts compared with the day 12 cell numbers (Supplemental Figure 5).

### CD3^+^γδTCR^+^IL-23R^+^ are necessary and sufficient to drive IL-23–mediated inflammation.

These data support that tissue-resident T cells respond to IL-23 in the local ocular environment to elicit inflammation (uveitis). To test the dependence of uveitis on T cells, we went on to identify whether Th cells (Th17) or Tγδ17 or other cell types such as ILC3 were the central drivers for the early response to localized IL-23 expression. All these cell lineages express functional IL-23 receptors (24, 29, 55), but unlike Th17 or Tγδ17 T cells, ILC3 do not require Rag2 expression for their development (67).

**Figure 4:**
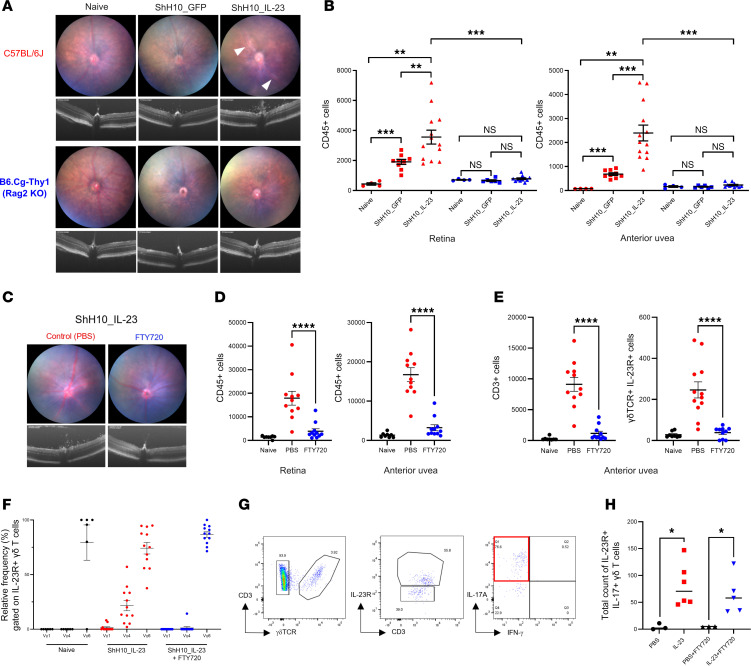
CD3^+^γδTCR^+^IL-23R^+^ are both necessary and sufficient to drive IL-23–mediated inflammation. C57BL/6J and B6.Cg-Thy1 (Rag2-KO) received intravitreal injection of 1 × 10^11^ vector genomes of ShH10_IL23 or ShH10_GFP vectors. Clinical monitoring (fundus and OCT) performed until day 12. Eyes were enucleated and prepared for flow cytometry to quantify immune cell infiltration in the anterior uvea and retina. (**A**) Representative clinical images show perivascular sheathing (white arrowheads) and vitreous infiltrate present in C57BL/6J eyes receiving ShH10_IL23 only. (**B**) Total live CD45^+^ cell counts (anterior uvea or retina) from C57BL/6J or Rag2-KO mice at day 12 (naive [*n* = 4]; ShH10_GFP [*n* = 6–9] or ShH10_IL-23 [*n* = 6–13]; data combined from 2 independent experiments). IL-23R–eGFP (+/–) mice were bilaterally injected with ShH10_IL23 (1 × 10^11^ vector genomes) and allocated to groups (*n* = 6) for repeated oral dosing with fingolimod (FTY720; 10 mg/kg) or vehicle control, administered on alternate days following AAV injection. On day 12, eyes were evaluated using clinical imaging and enucleated for flow cytometric analysis of cell infiltrate. (**C**) Representative images show inflammation absent in eyes receiving FTY720 treatment. (**D** and **E**) Total live CD45^+^ counts in the anterior uvea and retina, and CD3^+^ and γδTCR^+^IL-23R^+^ cells in the anterior uvea. (**F**) Relative frequency of γδTCR^+^ expressing Vγ1, Vγ4, or Vγ6 in naive (*n* = 6) and ShH10_IL23 injected eyes receiving vehicle or FTY720 treatment (*n* = 12). (**G** and **H**) Following ex vivo restimulation, representative gating of anterior uvea γδTCR^+^IL-23R^+^ cells in response to ShH10_IL23 to show relative frequency of IL-17A–expressing cells is equivalent between the FTY720 and vehicle treatment groups. Statistical analysis was performed with 1-way ANOVA. Data expressed as means ± SEM. ns = not significant. ** = *P* < 0.01, *** = *P* < 0.001 and **** =*P* < 0.0001 (**C**–**E**). Mann-Whitney test; * = *P* < 0.05 (**H**).

C57BL/6J and B6.Cg-Thy1 (Rag2-KO) mice, receiving 1 × 10^11^ vg ShH10_IL-23 in one eye and control ShH10_GFP vector in the contralateral eye were clinically monitored until day 12; cellular infiltrate in the anterior and posterior compartments were assessed by flow cytometry. In Rag2-deficient animals, administration of the ShH10_IL-23 (or control ShH10_GFP) did not replicate any of the clinical inflammatory changes (perivascular sheathing, vitreous infiltrate) that are observed with IL-23 overexpression in WT C57BL/6J mice (Figure 4A). The absence of clinical inflammation in the Rag2 mice is confirmed by flow cytometry analysis, demonstrating no recruitment of CD45^+^ infiltrating cells to either ocular compartment (Figure 4B). These data suggest that T cells (Th17 or Tγδ17) are the key effector cell type responding to IL-23 in the eye.

Recognizing the primed effector phenotype and pathogenic capacity of the resident IL-23R^+^γδ^+^ T cells in healthy tissue, we next explored how local IL-23 expression influences effector function (IL-17A production) and the recruitment of peripheral immune cells. IL-23R–eGFP reporter mice injected with ShH10_IL23 or vehicle (PBS) in the contralateral eye were treated with a repeated oral dosing regimen of FTY720 (10 mg/kg), a potent Sphingosine-1-phosphate receptor 1 (S1PR1) agonist that hinders T cell migration to inflammatory sites including the eye (68). At day 12 after AAV injection, no clinical disease or CD45^+^ infiltrate was evident, confirming that FTY720 treatment inhibits the recruitment of peripheral immune cells to both the retina and anterior uvea in response to local IL-23 expression (Figure 4, C and D). Analysis of the anterior uvea highlights that, as FTY720 inhibitory action prevents the increase in total CD3^+^ number, the resident CD3^+^IL-23R^+^γδ^+^ T cell population size remains comparable with the naive tissue. In contrast, absence of S1PR1 blockade leads to a reciprocal increase in the number of CD3^+^IL-23R^+^γδ ^+^ cells (Figure 4E), as a mixed population of cells comprising Vγ1, Vγ4, and Vγ6 subsets (Figure 4F). Following ex vivo stimulation, IFN-γ expression was not detected (Figure 4G), with the frequency of IL-23R^+^γδ^+^IL-17A^+^ cells similar between the vehicle and FTY720 treatment groups (Figure 4H).

The response to IL-23 exposure confirms an increase in Tγδ17, which accompanies the recruitment of αβ IL-17^+^ T cells to the anterior uvea during active inflammation (Supplemental Figure 6, A and B). A significant influx of αβ IFN-γ^+^ T cells was also observed, becoming the prevalent infiltrating population (Supplemental Figure 6, C and D). Importantly, the majority of infiltrating αβ T cells do not express IL-23R, and indicates the resident γδ T cells are the primary IL-23R^+^IL-17^+^ population in the eye following IL-23 stimulation (Supplemental Figure 7).

### Human anterior uvea contains CD3^+^γδTCR^+^ cells capable of producing IL-17A.

Our data from the mouse demonstrates that the anterior uvea contains resident CD3^+^IL-23R^+^γδ^+^IL-17A^+^ T cells that, when activated, drive the recruitment of peripheral CD45^+^ immune cell populations and promote inflammation in both ocular compartments. To understand whether a similar resident population could be identified in humans, we next characterized the immune cell biology in the equivalent insertional regions of the human eye using postmortem tissue obtained from healthy donors. Confocal microscopy reveals CD3^+^ T cells are present in key structural areas of the anterior uvea (AU) including the iris, ciliary body, ICA and the limbal sclera as far as the peripheral cornea (Supplemental Figure 8).

**Figure 5:**
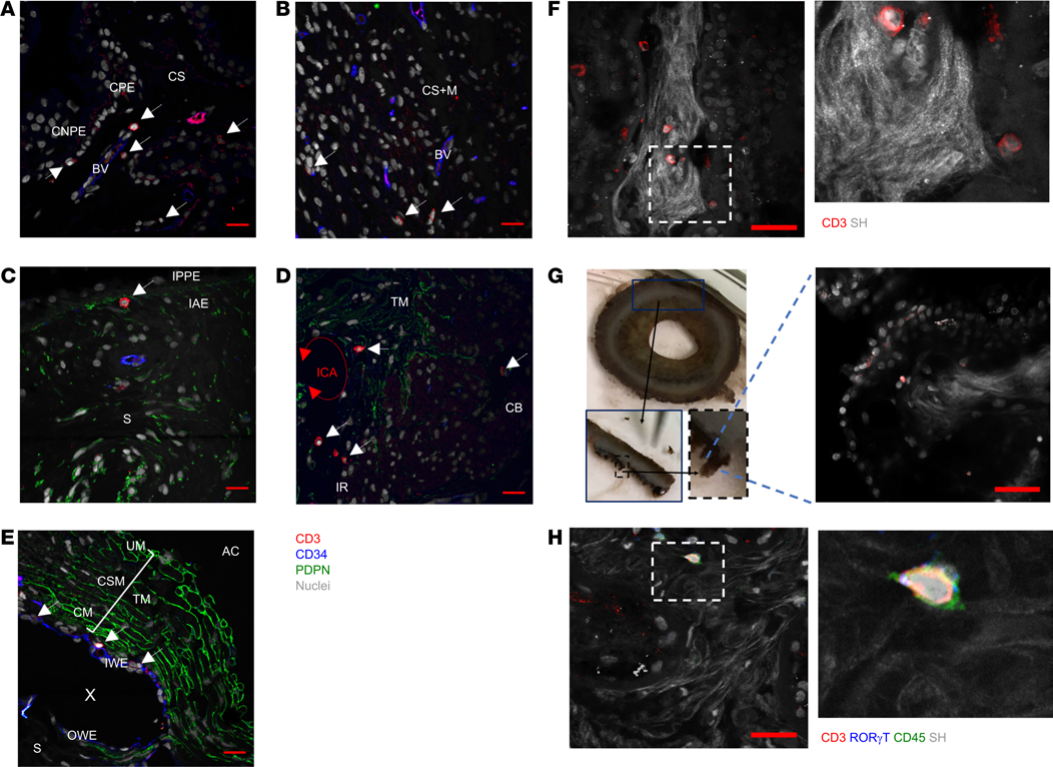
The human anterior uvea contains tissue resident CD3^+^ cells. FFP tissue sections prepared from an enucleated human eye with a healthy anterior chamber were costained with antibodies to CD3 (red), CD34 (blue), podoplanin (green), and Hoechst nuclear stain (gray). (**A**–**E**) Representative confocal immunofluorescence (IF) images showing extravascular location of CD3^+^ T cells in the ciliary processes (**A**); ciliary body proximal to the longitudinal ciliary muscles (**B**); within the ciliary region of the iris proximal to the anterior epithelium and dilator muscles of the posterior border layer (**C**); at the border of the ICA and arranged alongside PDPN+ trabeculae cells (**D**); and in the cribriform layer of the trabecular meshwork (TM) adjacent to the inner wall of Schlemm’s canal (**E**). AC, anterior chamber; BV, blood vessel; CNPE, ciliary nonpigmented epithelium; CPE, ciliary pigmented epithelium; CS, ciliary stroma; CS+M, ciliary stroma and muscle; IAE, iris anterior epithelium; IPPE, iris pigmented epithelium; IR, iris root; ICA and red arrowheaded line, iridocorneal angle; CM, cribriform meshwork; IWE, inner wall epithelium of Schlemm’s canal; OWE, outer wall epithelium of Schlemm’s canal; X, the lumen of Schlemm’s canal. Scale bar: 20 mm. (**F** and **G**) Representative immunofluorescence image showing merged expression of second harmonic resonance (SHR) collagen fibers and nuclei (gray), and CD3 (red) within a major ciliary process. Hashed squares highlight magnified region. Photographs detailing dissection of the major ciliary process from healthy human anterior uvea for whole mount staining, with SHR image showing merged expression of Col IV and CD3. Scale bar: 50 μm. (**H**) Images to show merged expression of SHR collagen fibers, CD3, and RORγt (blue). Hashed square highlights magnified region. All SHR images captured at ×40 magnification.

To determine whether these T cells were in the eye tissue and not intravascular artifact, eye tissue sections were costained with CD34 and podoplanin (PDPN), markers of vascular and lymphatic endothelium, respectively. This confirmed that tissue-resident T cells are located within the folds of the ciliary processes (Figure 5A); ciliary body proximal to the ciliary muscles (Figure 5B); ciliary region of the iris, proximal to the anterior epithelium and dilator muscles of the posterior border (Figure 5C); the border of the ICA, arranged alongside PDPN^+^ trabeculae cells (Figure 5D); and the cribriform layer of the TM adjacent to wall of Schlemm’s canal (Figure 5E). No T cells were observed in the uveal meshwork or corneoscleral meshwork except where trabeculae merge with the iris root and penetrate the ciliary body at the ICA.

To assess the relationship of CD3^+^ cells with the collagenous vascular core of the ciliary processes, and the basement membrane of the ciliary pigmented epithelium (CPE), high-magnification multiphoton imaging was utilized to generate second harmonic resonance images of the collagen fibers from coronal sections of healthy tissue (Figure 5F). This demonstrates that T cells are closely associated with the fibers that constitute the dense collagen core of the ciliary processes. Most T cells were observed at the center of the collagenous cores, but cells are also located proximal to fine collagen fibers, inserting into the CPE. This was validated by whole mount imaging of ciliary body tissue to negate the potential for displacement of T cells during the sectioning process (Figure 5G). Despite low frequency, second harmonic imaging also enabled us to identify RORγt^+^ expression of CD3^+^ cells within the normal ciliary body (Figure 5H).

**Figure 6:**
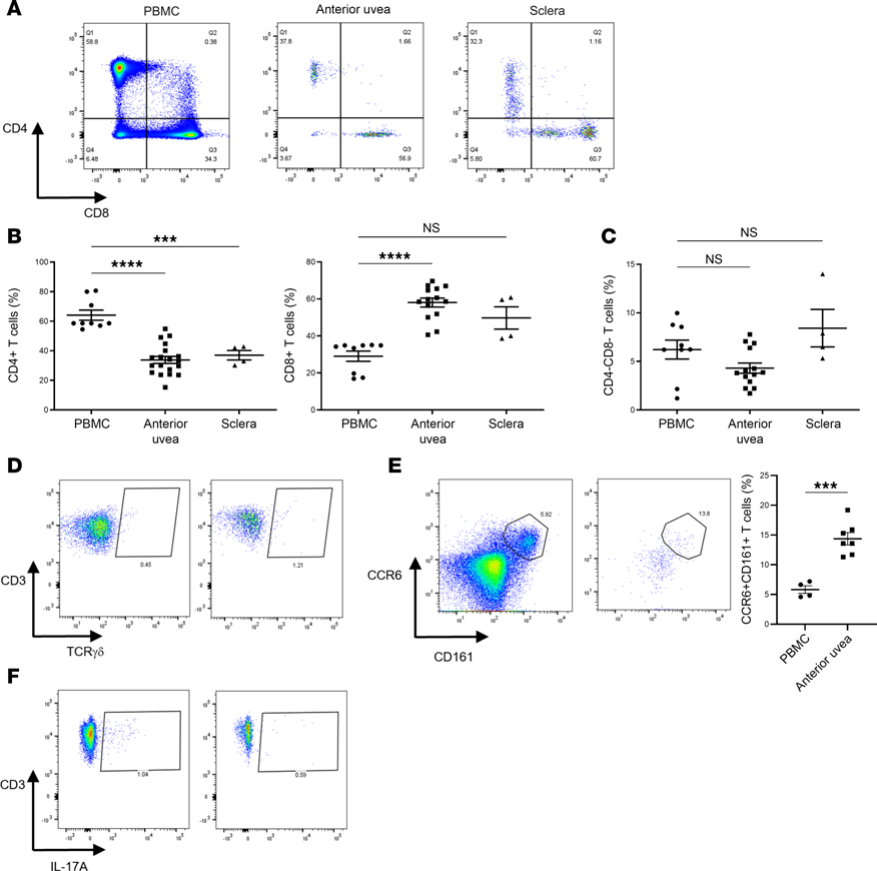
CD3^+^γδTCR^+^ cells in the anterior uvea and sclera can produce IL-17A. Flow cytometric analysis was used to phenotype T cell subsets from postmortem anterior uvea (AU) and sclera (S) tissue samples and compared with peripheral blood mononuclear cells (PBMCs) isolated from normal healthy controls. (**A** and **B**) Representative FACS plots showing CD3^+^ populations positive for CD4^+^ or CD8^+^ isolated from AU, S. and PBMCs and compiled frequency data for the percentage of different CD3^+^ T subsets. (**C** and **D**) Relative frequency of CD3^+^CD4^–^CD8^–^ cells observed in ocular tissues (AU and S) and PBMCs, and representative FACS plots to show CD3^+^γδTCR^+^ cells in AU and S. Samples: anterior uvea (*n* = 19; data obtained from 7 independent experiments), sclera (*n* = 4; data obtained from 2 independent experiments), and control blood (*n* = 9; data obtained from 4 independent experiments). Mean percentage is represented by the central line with data shown as mean ± SEM. ***P < 0.001 and ****P < 0.0001 (Brown-Forsythe and Welch ANOVA, with Dunnett’s T3 correction for multiple comparisons). (**E**) Representative FACS plots to demonstrate gating of CCR6^+^CD161^+^ cells in PBMC and AU samples, and graph showing relative frequency of this population within the CD3^+^ T cell pool. Samples: PBMC (*n* = 4) AU (*n* = 7). ***P < 0.001 (unpaired Student’s 2-tailed *t* test). (**F**) FACS plots of AU and S following in vitro stimulation with PMA/Ionomycin to demonstrate a fraction CD3^+^ cells express IL-17A.

To exclude the potential artifact of peripheral blood contamination, we compared the relative frequencies of T cell subsets present in postmortem AU and scleral (S) tissue, with peripheral blood mononuclear cells (PBMCs) isolated from normal healthy controls (Figure 6A and Supplemental Figure 9, A and B). In comparison with blood, AU and S samples contain a significantly lower proportion of CD4^+^, with a higher frequency of CD8^+^ (Figure 6B). Flow cytometry highlights AU and S tissues possess a diverse array of leukocyte subsets, including CD3^+^CD4^–^CD8^–^ T cells (Supplemental Figure 9, C and D). While the relative frequency of this population remains unchanged compared with PBMCs (Figure 6C), a small subset of CD3^+^ T cells were γδTCR^+^ (Figure 6D). Extended immune-phenotyping of the CD3^+^γδTCR^+^ population compared with PBMCs revealed a higher frequency of AU CD3^+^ T cells expressing CCR6 and CD161 (Figure 6E), producing IL-17A following ex vivo stimulation (Figure 6F).

Collectively, these human data reveal that the healthy anterior tissues contain populations of T lymphocytes that are located within collagenous cores at the center of the ciliary processes, within the ciliary body and the sclera.

## Discussion

Here we present evidence that characterizes a resident population of IL-23R^+^ γδ T cell, primed to rapidly respond to IL-23 exists within the anterior uvea in mice and humans. Consistent with previous reports of the functional presence of γδTCR^+^IL-23R^+^ T cells at MSK entheseal tissues and aortic root (21, 28), CD3^+^CD4^–^CD8^–^CD69^+^γδTCR^+^IL-23R^+^ T cells also constitute the majority of resident anterior compartment IL-23R^+^ T cells. Despite being present at a low frequency in normal healthy tissue, these cells exhibit a “primed” phenotype with an intrinsic capacity to secrete IL-17A as pathogenic effectors. In vivo, localized induced ocular expression of the IL-23 cytokine demonstrates that resident CD3^+^γδTCR^+^IL-23R^+^ cells are both required and sufficient following activation to drive the recruitment of peripheral CD45^+^ infiltrating cells and promote inflammation (uveitis) in the mouse eye. Furthermore, data from study of human anterior uvea tissue demonstrate extravascular location of resident CD3^+^γδTCR^+^ cells with capacity to generate IL-17A, identifying potential IL-23R–mediated responsiveness in man.

The healthy uvea (anterior and posterior regions) of the eye have traditionally been considered devoid of lymphocytes, outside circulating cells in blood vessels (69), with occasional studies reporting the occurrence of conventional αβ TCR^+^ T cells in the normal iris and ciliary body (70, 71). Understanding of tissue-resident lymphocyte populations has expanded over the past few years, and resident memory T cells (T_RM_), as well as other cell types including ILCs and nonclassical T cells expressing unique TCR heterodimers (γδ T cells), have been identified (72, 73). Typically, high numbers of γδ T cells are found at barrier surfaces, including the conjunctiva (lining of the eyelid and globe), which possesses a resident population involved with regulating mucosal immunity and barrier homeostasis in host defense (74). Recent reports using a transgenic TCR reporter mouse suggest the presence of γδ T cells in tissues proximal to the limbal sclera (without any reference to vasculature) (28) and present within the choroidal vasculature of healthy human donor eyes (75). Taken together, this supports our data that T cells are present in the uvea.

Using nonpigmented (albino) and IL-23R reporter transgenic naive mice, we demonstrate the anatomical location and phenotype of this potentially novel resident T cell population present in the healthy anterior uvea and adjacent tissue. Imaging the whole intact anterior compartment revealed clusters of CD3^+^ cells located within the peripheral cornea, sclera, and ciliary body. Immunofluorescence on tissue sections from IL-23R–eGFP mice pinpoints their extravascular niche by demonstrating that cells reside within the pars plicata of the ciliary body, proximal to longitudinal ciliary muscle and within the folds proximal to the ciliary epithelium, the iris, and stromal layer of the adjacent sclera. The ciliary body, as a circular muscle positioned immediately behind the iris, functions to produce ocular aqueous fluid but is also connected via zonular fibers enabling changes of crystalline lens shape (accommodation). Furthermore, this region of the anterior compartment is also proximal to extraocular muscle insertions. Accordingly, these intra- and extraocular tissues are intimately involved with mechanical movement and stress and are analogous to the MSK entheses; we therefore consider them as ocular entheses.

To enhance deeper immune-phenotyping of this rare cell population within the naive eye, we utilized multiparameter/spectral flow cytometry techniques. Enzymatic digestion to liberate immune cells revealed the healthy mouse anterior uvea contains a relatively small population of CD3^+^CD4^–^CD8^–^CD69^+^γδTCR^+^IL-23R^+^ T cells. Importantly, the γδTCR^+^IL-23R^+^ population exhibits a “primed” phenotype, confirmed through surface expression of (CD44^hi^CD62L^–^CD27^–^CCR6^+^), and intracellular expression of RORγt, which drives the secretion of proinflammatory IL-17A upon stimulation. These cells share characteristics of pathogenic effectors, including the same steady-state phenotype as the γδ T cell populations described at the entheseal regions of the axial skeleton and aortic root in the mouse (21, 28).

γδ T cells are a unique T cell subpopulation, rare in secondary lymphoid organs but enriched in many peripheral tissues with an intrinsic capacity to express large amounts of effector cytokines (IFN-γ or IL-17A) that shape local immune responses (76). Different waves of γδ T cell progenitor subsets, classified based on their somatic T cell receptor gene rearrangements (TCRγ; Vγ chain) and functional potential are produced during specific developmental windows in the thymus before selectively homing to different organs (50, 77–79). While the extent of their functional roles is still being defined, homing to peripheral sites is widely considered to provide a mechanism that expands spatial immune responsiveness in tissues not served by conventional αβ T cells. In addition to immune surveillance, recent studies highlight other roles in steady-state physiology with evidence of involvement in neuronal synaptic plasticity in the CNS (80).The γδTCR^+^IL-23R^+^ population identified in the normal mouse anterior tissues display Vγ6 TCR homogeneity (no expression of Vγ1 or Vγ4), combined with their primed surface phenotype and cytokine profile, indicating that these cells represent a long-lived IL-17–producing γδ T cell population similar to the skin, entheseal tissues, lung, reproductive tract, and brain (51, 52, 79). Elaboration of the embryonic development window and timing when these cells are seeded in the anterior eye tissue will help inform as to whether this resident population exert other physiological roles, akin to CNS γδ T cells. The current characterization was undertaken in adult mice (6–8 weeks), therefore examining the extent of their resident tissue longevity, alongside the influence of age-associated decline in their functional immune phenotype in the anterior uvea, will be important. Aging leads to substantial compositional changes in the peripheral lymph node γδ T cell pool in mice, skewed toward an expansion and polarization of cells toward IL-17–producing Vγ6 γδ T cells alongside age-related increases in tumor incidence (81). In humans, an altered γδ T cell usage and increased effector phenotype is observed with age (82). If similar is true for tissue-resident populations in the anterior compartment and tissues associated with AxSpA, such age-related changes may explain the link to increased risk for extra-articular manifestations in AxSpA, including AAU.

To understand how resident γδTCR^+^IL-23R^+^ cells could act as pathogenic effectors in the context of AAU, we employed localized ocular IL-23 overexpression, previously achieved through systemic DNA minicircle injection which reported evidence of AU inflammation, including accumulation of γδTCR^+^ cells adjacent to the ciliary body (21, 28, 29, 83). Development of the ShH10-IL-23 AAV vector for intravitreal delivery facilitated ocular restricted expression of IL-23 to interrogate the role of the resident population in driving inflammation. Inflammation elicited in the mouse eye replicates many clinical features that correlate to human disease, namely posterior (retina) inflammation, and cellular infiltration in both the vitreous and aqueous compartments. Following injection, constitutive secretion of IL-23 led to rapid onset of inflammation, followed by a chronic and persistent disease phenotype not observed or attributed to ShH10_GFP response. Use of AAV to model, probe, and evaluate therapeutics for human inflammatory ocular disease is an expanding area (84, 85) and, in the current context, provides a tool to interrogate pathogenicity of IL-23 alone in a disease that is accompanied by elevated concentrations of serum and AqH IL-23 (41, 43, 44, 86).

Taking an iterative approach to highlight the effector function of the rare population of T cells, we deployed Rag2 deficiency and S1PR1 antagonism (fingolimod treatment), which unequivocally demonstrated the resident CD3^+^γδTCR^+^IL-23R^+^ population and not other cells types such as ILC3, and found that it is both necessary and sufficient to drive uveitis. FACS demonstrates IL-23 expression correlates to the level of immune cell infiltrate, with CD45^+^ populations comprising elevated numbers of CD3^+^ T cells (both CD4^+^ and CD8^+^), and recruitment of monocytes, macrophages, dendritic cells, B cells, and neutrophils.

Anterior eye tissue contained significantly higher numbers of CD3^+^ γδTCR^+^ T cells, despite the relative frequency of cells exhibiting the preactivated effector phenotype in response to IL-23 remaining unchanged when compared with either control AAV injected or naive eyes. Our interpretation was that the resident population lacks proliferative capacity in response to IL-23 stimulation, leading us to consider whether the expanded number was due to peripheral recruitment. Extended phenotyping of CD3^+^γδTCR^+^IL-23R^+^ cells support this hypothesis, revealing a heterogeneous γδ T cell pool comprising Vγ1, Vγ4, and Vγ6 subsets. Furthermore, evidence that corroborates their recruitment following activation is shown in analysis of anterior tissues taken from the mice receiving fingolimod intervention (pharmacological-induced lymphopenia), where a normal frequency (equivalent to naive) and homogeneity Vγ6^+^ γδ subset is maintained. We speculate that the most likely origin of the Vγ1 and Vγ4 cells recruited to the eye are from local peripheral lymphoid organs, as these tissues are the recognized seeding location of these subsets during the post-natal wave of immune development (79). Further approaches to delineate the γδ T cell phenotype and composition within the local draining lymph nodes, including transcriptomic and cell tracking studies, would be informative.

Nonetheless, these resident cells do not secrete IFN-γ following ex vivo stimulation, emphasizing their functional capacity at this location as γδ IL-17–producing (Tγδ17) cells that can orchestrate and promote tissue inflammation. While Tγδ17 are considered exclusively derived from foetal thymus, studies indicate they undergo homeostatic proliferation and self-renewal in peripheral tissues to maintain their numbers (77, 81, 87, 88). Furthermore, under specific conditions including TCR stimulation and the presence of IL-1β and IL-23 cytokines, Vγ4^+^ γδ17 T cells also have the potential to develop and expand in the adult mouse (89).

The observations of IL-23R–mediated responsiveness of a previously unknown resident γδ T cell population in the mouse, raises the exciting potential that a similar population also exists in humans. In this study, we provide early exploratory evidence that CD3^+^γδTCR^+^ cells with capacity to secrete proinflammatory IL-17A exist at equivalent insertional regions of the human eye. Tissue sections highlight CD3^+^ cells located throughout the anterior uvea, including the iris, TM, sclera, and the ciliary body, intimately associated with collagen-rich core of the ciliary processes. The higher CD8/CD4 ratio observed in the human AU and sclera is consistent with another unpublished study that defines T cell subpopulations, including multiple clusters of CD8^+^, CD4^+^, and double-negative γδ T cells in noninflamed and uveitic human anterior uvea (90). It provides new evidence of conventional long-lived CD3^+^CD8^+^CD69^+^CD103^+^ T_RM_ but also TCR-Vd1^+^ IL-23R^+^ cells reside, and are enriched during active disease.

Despite the low detectable frequency on tissue sections examined, second harmonic tissue imaging identified CD3^+^RORγt^+^ coexpression. However, recognizing the challenges associated with tracking IL-23R and RORγt expression in human cells, our FACS phenotyping instead evaluated CCR6 and CD161 expression on CD3^+^ cells obtained from anterior tissues (91). Further work to attribute IL-23R responsiveness and comprehensive phenotype of the different resident T cell populations that we now recognize to exist within the human anterior compartment is required, as single-cell multi-omics datasets provide evidence of lymphocyte populations within the ciliary body (92, 93). In the future, approaches harnessing transcriptomic and enhanced immunophenotyping will provide clarity on whether the putative IL-23R^+^ γδ T cells observed in mouse also exist in humans (90). In support, emerging evidence from single-cell RNA-Seq of aqueous biopsies taken from uveitis patients reveals that infiltrates in HLA-B27–associated uveitis are dominated by unconventional T cells (γδ T cells) and myeloid cells (94).

In summary, using murine models we have shown that anatomical sites that are typically inflamed in AAU are primed to rapidly react to IL-23 by the presence of this previously unidentified population of IL-23R^+^ resident cell. In vivo exposure to IL-23 is sufficient to induce highly specific ocular inflammation in the absence of Th17 cells and with rapid kinetics. We propose that the ocular enthesis is therefore a functional IL-23–responsive anatomic site, similar to the MSK entheseal tissues, gut, and lung, which also contain innate IL-23R expressing cells (95), primed to respond immediately to IL-23. In the context of AxSpA related AAU, promising results using IL-17 antagonists (Secukinumab & Ixekizumab) or combined IL-12/23 biologics (Ustekinumab) suggests modulating these pathways in patients requiring long-term control can provide avenues to intervene for both disease manifestations (96, 97).

## Methods

### Sex as a biological variable

Equal numbers of sex-matched mice were used both for characterization of naive tissue and in disease (involving ShH10_IL23 administration) experiments.

### Animals

Adult C57BL/6J were purchased from Charles River Laboratories, UK. B6(Cg)-Tyrc-2J/J (Albino), B6.Cg-Thy1 (recombination activation gene (Rag)2 KO), and IL-23R–eGFP mice (on C57BL/6J and B6(Cg)-Tyrc-2J/J backgrounds) were supplied from established breeding colonies at the University of Bristol. Homozygous IL-23R–eGFP^GFP/GFP^ mice (55) were mated with C57BL/6J or B6(Cg)-Tyrc-2J/J mice to generate heterozygous reporter mice for experiments. All mice were used at 6–12 weeks of age. All mouse strains were confirmed as negative for the Rd8 mutation (98) and were housed under specific pathogen free conditions with food and water ad libitum.

### Intravitreal injection, in vivo imaging and treatment interventions

Prior to any procedure, pupils were dilated using topical tropicamide 1% w/v and phenylephrine 2.5% w/v (Minims; Chauvin Pharmaceuticals), before anaesthesia with 2% isofluorane (Piramal Critical Care). AAV was administered by intravitreal injection (2 μL/eye), using a 33G needle on a microsyringe under direct visualization (Hamilton Company). Retina fundal imaging and optical coherence tomography (OCT) scans were captured using Micron IV (Phoenix Research Laboratories). Clinical scoring using vitreous cell densitometry was performed on circular retinal OCT images centerd on the disc using ImageJ software (NIH) (99).

For FTY20 treatments, mice were orally dosed with FTY720 (fingolimod-hydrochloride; Caymen Chemicals) 10mg/kg or vehicle control, administered on alternate days following AAV injection. Mice were assigned to treatment groups (FTY720 or vehicle) in a constrained randomized order within blocks, dependent on cage allocations and litter they were derived from.

### Flow cytometry

### Murine ocular tissue

#### Dissection and dissociation.

Following enucleation, single eyes were dissected in 100 μL of ice-cold PBS. Using a limbal incision the posterior segment was removed, whole retina and vitreous extracted and together with the dissecting fluid (PBS) transferred into a 1.5 mL Eppendorf tube. The tissue was mechanically dissociated by rapping the tube across an 80-well standard rack 10 times.

For anterior tissue, following lens removal, the iris, ciliary body and limbal sclera was first mechanically dissociated in a culture dish using scissors, before enzymatic digestion in 0.5mL DMEM containing 5 mg/mL type II collagenase (Worthington, LS004202) and 0.2 mg/mL DNase I (Roche, 11284932001) for 45 minutes at 37°C undergoing constant, gentle agitation. The enzymatic digest was stopped by adding 0.5mL of complete media (DMEM containing 10% FCS), centrifuged at 250*g* for × 10 minutes and cell pellets resuspended in 100ul of cold PBS.

Retina and anterior cell suspensions were transferred into a 96-well 60 mm cell filter plate (Merck Millipore) and washed with 150 μL of PBS. The plate was centrifuged at 1200 rpm for 5 minutes, the supernatant aspirated and cells resuspended in 0.1% bovine serum albumin (BSA) fluorescence-activated cell sorting buffer and transferred into a 96-well V-bottom plate for immunostaining.

#### Cell surface marker staining.

Cells were incubated with purified rat anti-mouse CD16/32 Fc block (1:50; 553142, [2.4G2], BD) for 10 minutes at 4°C before incubation with fluorochrome-conjugated monoclonal antibodies against mouse cell surface markers (Supplemental Table 2) at 4°C for 20 minutes. Cells were washed and resuspended in 7-aminoactinomycin D (Thermo Fisher Scientific) for dead cell exclusion.

#### Intracellular cytokine staining.

Anterior uvea or retinal cell suspensions were initially stimulated for 2 hours with 50 ng/mL phorbol myristate acetate (PMA) and 500 ng/mL Ionomycin, and then for a further 2 hours with the addition of 1 mg/mL Brefeldin and Monensin (BD Biosciences) in complete medium. Cells were fixed, permeabilized with the Cytofix/perm solution (BD Biosciences), and stained with intracellular cytokine antibodies (Supplemental Table 3) LIVE/DEAD fixable cell staining kit (Thermofisher Scientific) was used to exclude dead cells from analysis.

#### Cell acquisition.

Cell suspensions were acquired using a fixed and stable flow rate for 2.5 minutes on a 4-laser Fortessa X20 flow cytometer (BD Cytometry Systems). Compensation was performed using OneComp eBeads (01-1111-41, Thermo Fisher Scientific). Seven 2-fold serial dilutions of a known concentration of splenocytes were similarly acquired to construct a standard curve and calculate absolute cell numbers (100). Analysis was performed using FlowJo software (Treestar).

#### ImageStream analysis.

Following surface receptor staining, anterior uvea cell suspensions were fixed, permeabilized using the FOXP3 staining kit (Ref: 00-5523, eBioscience) and stained with RORγt (1:40; 12-6981-82, [B20] eBioscience). Cells were washed with PBS containing 2% FCS twice. From naive eyes, x4 anterior uvea cell suspensions were pooled, and image-based flow cytometry analysis conducted in Oxford using the ImageStream MkX. DAPI (2 μg/mL; BD Pharmingen; 564907) was added to samples immediately prior to acquisition. 10,000 events per sample were collected and analyzed using the IDEAS software.

### Human ocular tissue

Human donor eye material (partial globes) were supplied, transported and processed within 36 hours from time of death. Patients with known inflammatory or infectious condition of the entheses were excluded from the study.

#### Dissection and cell dissociation.

Briefly, the entire anterior uvea was removed by cutting around the circumference from the choroid to the base of the ciliary body (avoiding any sclera and retinal attachment from the ora serrata. Following this, a ~2 mm cut around the circumference of the limbal sclera was made (which included any muscular attachment sites). Both tissues were washed twice in Dulbecco’s Modified Eagle’s Medium (DMEM; Gibco, 41965-039), cut into small ~1mm^3^ pieces and enzymatically digested in DMEM containing 5 mg/mL type II collagenase (Worthington, LS004202) and 0.2 mg/mL DNase I (Roche, 11284932001) for 120 minutes at 37°C undergoing constant, gentle agitation. The digest was then washed with complete media and filtered twice through a 70 μm cell strainer (Corning, CLS431751-50EA). Single cell suspensions were stained with the Zombie NIR Fixable Viability KIT (780 nm of excitation; Biolegend, 423106) at a 1/250 dilution in PBS (Biolegend, 420201) for 20 minutes at 4°C.

#### Cell surface receptor staining.

Cells were washed (Cell staining buffer; Biolegend) before resuspension in Human TruStain FcX blocking agent, before incubation with fluorochrome-conjugated monoclonal antibodies against human cell surface markers (Supplemental Table 4) for 20 minutes on ice. Cells were washed twice then placed into 400 μL cell staining buffer in FACS tubes. Compensation controls were made using UltraComp eBeads (eBioscience). Cells were analyzed on a 3 laser (405nm, 488nm, and 640nm) BD LSR Fortessa X-20 flow cytometer (BD Biosciences). Medium or high flow rates were used. FACSdiva and FlowJo software (BD Biosciences) were used for analysis of data. Compensation was performed using OneComp eBeads (eBioscience, 01-1111-42).

#### Intracellular cytokine staining.

Intracellular cytokine staining of T-cells was performed by stimulating cell suspensions for 2 hours with 50 ng/mL phorbol myristate acetate (PMA) and 500 ng/mL Ionomycin then for a further 2 hours with the addition of 1 mg/mL Brefeldin and Monensin (all from Sigma Aldrich) in D10 medium. Following surface antigen staining, the Transcription Factor Buffer Set (BD Biosciences) was used according to the manufacturer’s protocol for intracellular staining. A specific dilution of each primary conjugated antibody was used (Supplemental Table 5). Following this, cells were washed twice then placed into 200 μL 0.1% w/v BSA in PBS in FACS tubes.

### Statistics

Statistical tests applied and *n* sample numbers are detailed in the figure legends. Data were analyzed using GraphPad Prism software (v9.4). Variance was compared using an F-test. Data with equal variances were analyzed using the unpaired Students 2-tailed *t* test. Nonparametric data (e.g., ocular cell infiltrate) were analyzed using 1-way ANOVA and Tukey’s multiple-comparison test, with data expressed as means ± SEM. In the analysis of human PBMC, anterior uvea and sclera T cell subsets flow cytometry, groups were analyzed using the Brown Forsythe & Welch ANOVA, with Dunnett’s T3 correction for multiple corrections. *P* < 0.05 was considered statistically significant.

### Data availability

All relevant information about data is available directly from the corresponding author. Values for all data points in graphs are reported in the Supporting Data Values file.

### Study approval

All procedures were conducted in concordance with the United Kingdom Home Office license (PPL PP9783504) and were approved by the University of Bristol Ethical Review Group. The study also complied with the Association for Research in Vision and Ophthalmology (ARVO) Statement for Use of Animals in Ophthalmic and Vision research.

Human donor eye material surplus to corneal transplantation (without recorded ocular disease) was obtained from National Health Service (NHS) Blood and Transplant Services after research ethics committee approval (REC#: 07-H0706-81), with experiments conducted according to the Declaration of Helsinki and in compliance with UK law.

## Author contributions

JS and ADD conceived the study. RH, AW, CJC, and SGD designed, performed, and or analyzed experiments. SEC provided samples of consented human tissue. DAC, JS, ADD, SK, and PCT designed and or analyzed experiments and supervised research. RH, CDB, JS, ADD, and DAC wrote the manuscript, with contributions from all other authors. RH and AW contributed equally to this work and are co–first authors. Data was presented and discussed within wider ORBIT Consortium members.

## Supplementary Material

Supplemental data

Supplemental video 1

Supporting data values

## Version 1 08/28/2025

In-Press Preview

## Version 2 10/08/2025

Electronic publication

## References

[1] Taurog JD (2016). Ankylosing spondylitis and axial spondyloarthritis. N Engl J Med.

[2] Bridgewood C (2018). Spondyloarthritis: new insights into clinical aspects, translational immunology and therapeutics. Curr Opin Rheumatol.

[3] Moll JM (1974). Psoriatic arthritis: a unique family. Rheumatol Rehabil.

[4] Rosenbaum JT, Rosenzweig HL (2012). Spondyloarthritis: the eyes have it: uveitis in patients with spondyloarthritis. Nat Rev Rheumatol.

[5] Gracey E (2020). Revisiting the gut-joint axis: links between gut inflammation and spondyloarthritis. Nat Rev Rheumatol.

[6] Mielants H (2005). Gut inflammation in the spondyloarthropathies. Curr Rheumatol Rep.

[7] Harbord M (2016). The first European evidence-based consensus on extra-intestinal manifestations in inflammatory bowel disease. J Crohns Colitis.

[8] Brewerton DA (1973). Ankylosing spondylitis and HL-A 27. Lancet.

[9] Caffrey MF, James DC (1973). Human lymphocyte antigen association in ankylosing spondylitis. Nature.

[10] Schlosstein L (1973). High association of an HL-A antigen, W27, with ankylosing spondylitis. N Engl J Med.

[11] Burton PR (2007). Association scan of 14,500 nonsynonymous SNPs in four diseases identifies autoimmunity variants. Nat Genet.

[12] Cortes A, Brown MA (2011). Promise and pitfalls of the Immunochip. Arthritis Res Ther.

[13] Cortes A (2013). Identification of multiple risk variants for ankylosing spondylitis through high-density genotyping of immune-related loci. Nat Genet.

[14] Ellinghaus D (2016). Analysis of five chronic inflammatory diseases identifies 27 new associations and highlights disease-specific patterns at shared loci. Nat Genet.

[15] Evans DM (2011). Interaction between ERAP1 and HLA-B27 in ankylosing spondylitis implicates peptide handling in the mechanism for HLA-B27 in disease susceptibility. Nat Genet.

[16] Reveille JD (2010). Genome-wide association study of ankylosing spondylitis identifies non-MHC susceptibility loci. Nat Genet.

[17] Robinson PC (2016). Exome-wide study of ankylosing spondylitis demonstrates additional shared genetic background with inflammatory bowel disease. NPJ Genom Med.

[18] Bridgewood C (2020). Interleukin-23 pathway at the enthesis: The emerging story of enthesitis in spondyloarthropathy. Immunol Rev.

[19] Chan JR (2006). IL-23 stimulates epidermal hyperplasia via TNF and IL-20R2-dependent mechanisms with implications for psoriasis pathogenesis. J Exp Med.

[20] Yen D (2006). IL-23 is essential for T cell-mediated colitis and promotes inflammation via IL-17 and IL-6. J Clin Invest.

[21] Sherlock JP (2012). IL-23 induces spondyloarthropathy by acting on ROR-γt^+^ CD3^+^CD4^–^CD8^–^ entheseal resident T cells. Nat Med.

[22] Bridgewood C (2019). Identification of myeloid cells in the human enthesis as the main source of local IL-23 production. Ann Rheum Dis.

[23] Yawalkar N (2009). Increased expression of IL-12p70 and IL-23 by multiple dendritic cell and macrophage subsets in plaque psoriasis. J Dermatol Sci.

[24] Parham C (2002). A receptor for the heterodimeric cytokine IL-23 is composed of IL-12Rbeta1 and a novel cytokine receptor subunit, IL-23R. J Immunol.

[25] Cho ML (2006). STAT3 and NF-kappaB signal pathway is required for IL-23-mediated IL-17 production in spontaneous arthritis animal model IL-1 receptor antagonist-deficient mice. J Immunol.

[26] Geremia A (2011). IL-23-responsive innate lymphoid cells are increased in inflammatory bowel disease. J Exp Med.

[27] Watad A (2020). Normal human enthesis harbours conventional CD4^+^ and CD8^+^ T cells with regulatory features and inducible IL-17A and TNF expression. Ann Rheum Dis.

[28] Reinhardt A (2016). Interleukin-23-dependent γ/δ T cells produce interleukin-17 and accumulate in the enthesis, aortic valve, and ciliary body in mice. Arthritis Rheumatol.

[29] Buonocore S (2010). Innate lymphoid cells drive interleukin-23-dependent innate intestinal pathology. Nature.

[30] Jabs DA (2022). The standardisation of uveitis nomenclature (SUN) project. Clin Exp Ophthalmol.

[31] Thorne JE (2016). Prevalence of noninfectious uveitis in the United States: a claims-based analysis. JAMA Ophthalmol.

[32] Rothova A (1996). Causes and frequency of blindness in patients with intraocular inflammatory disease. Br J Ophthalmol.

[33] Gritz DC, Wong IG (2004). Incidence and prevalence of uveitis in Northern California; the Northern California Epidemiology of Uveitis Study. Ophthalmology.

[34] Rosenbaum JT (1989). Characterization of uveitis associated with spondyloarthritis. J Rheumatol.

[35] Bacchiega ABS (2017). Ocular involvement in patients with spondyloarthritis. Rheumatology (Oxford).

[36] Banares AA (1995). Bowel inflammation in anterior uveitis and spondyloarthropathy. J Rheumatol.

[37] Munoz-Fernandez S (2009). Enthesis inflammation in recurrent acute anterior uveitis without spondylarthritis. Arthritis Rheum.

[38] Juanola X (2016). Description and prevalence of spondyloarthritis in patients with anterior uveitis: The SENTINEL Interdisciplinary Collaborative Project. Ophthalmology.

[39] Haroon M (2015). A novel evidence-based detection of undiagnosed spondyloarthritis in patients presenting with acute anterior uveitis: the DUET (Dublin Uveitis Evaluation Tool). Ann Rheum Dis.

[40] Colbert RA (2010). From HLA-B27 to spondyloarthritis: a journey through the ER. Immunol Rev.

[41] Przepiera-Bedzak H (2016). Extra-articular symptoms in constellation with selected serum cytokines and disease activity in spondyloarthritis. Mediators Inflamm.

[42] Chi W (2007). IL-23 promotes CD4^+^ T cells to produce IL-17 in Vogt-Koyanagi-Harada disease. J Allergy Clin Immunol.

[43] Chi W (2008). Upregulated IL-23 and IL-17 in Behçet patients with active uveitis. Invest Ophthalmol Vis Sci.

[44] Jiang S (2010). Elevated serum IL-23 correlates with intraocular inflammation after cataract surgery in patients with Vogt-Koyanagi-Harada disease. Br J Ophthalmol.

[45] Jung JH (2017). The association between genetic polymorphisms of the interleukin-23 receptor gene and susceptibility to uveitis: a meta-analysis. BMC Ophthalmol.

[46] Dong H (2013). IL23R gene confers susceptibility to ankylosing spondylitis concomitant with uveitis in a Han Chinese population. PLoS One.

[47] Verstockt B (2023). IL-12 and IL-23 pathway inhibition in inflammatory bowel disease. Nat Rev Gastroenterol Hepatol.

[48] Jo SJ (2023). Efficacy of guselkumab in difficult-to-treat psoriasis regions: data from VOYAGE 1 and VOYAGE 2 Asian subpopulations. J Dermatol.

[49] Li W (2019). High-dimensional cell-level analysis of tissues with Ce3D multiplex volume imaging. Nat Protoc.

[50] Carding SR, Egan PJ (2002). Gammadelta T cells: functional plasticity and heterogeneity. Nat Rev Immunol.

[51] Prinz I (2013). Functional development of γδ T cells. Eur J Immunol.

[52] Heilig JS, Tonegawa S (1986). Diversity of murine gamma genes and expression in fetal and adult T lymphocytes. Nature.

[53] Zhou L (2007). IL-6 programs T(H)-17 cell differentiation by promoting sequential engagement of the IL-21 and IL-23 pathways. Nat Immunol.

[54] Lochner M (2008). In vivo equilibrium of proinflammatory IL-17^+^ and regulatory IL-10^+^ Foxp3^+^ RORgamma t^+^ T cells. J Exp Med.

[55] Awasthi A (2009). Cutting edge: IL-23 receptor gfp reporter mice reveal distinct populations of IL-17–producing cells. J Immunol.

[56] Sutton CE (2009). Interleukin-1 and IL-23 induce innate IL-17 production from gammadelta T cells, amplifying Th17 responses and autoimmunity. Immunity.

[57] Takatori H (2009). Lymphoid tissue inducer-like cells are an innate source of IL-17 and IL-22. J Exp Med.

[58] Sawa S (2010). Lineage relationship analysis of RORgammat^+^ innate lymphoid cells. Science.

[59] Singh SP (2008). Human T cells that are able to produce IL-17 express the chemokine receptor CCR6. J Immunol.

[60] Cibrian D, Sanchez-Madrid F (2017). CD69: from activation marker to metabolic gatekeeper. Eur J Immunol.

[61] Ribot JC (2009). CD27 is a thymic determinant of the balance between interferon-gamma- and interleukin 17-producing gammadelta T cell subsets. Nat Immunol.

[62] Qi C (2021). Gamma delta T cells and their pathogenic role in psoriasis. Front Immunol.

[63] Oppmann B (2000). Novel p19 protein engages IL-12p40 to form a cytokine, IL-23, with biological activities similar as well as distinct from IL-12. Immunity.

[64] Wu J (2020). Gene therapy for glaucoma by ciliary body aquaporin 1 disruption using CRISPR-Cas9. Mol Ther.

[65] Chan YK (2021). Engineering adeno-associated viral vectors to evade innate immune and inflammatory responses. Sci Transl Med.

[66] Tummala G (2021). Characterization of gene therapy associated uveitis following intravitreal adeno-associated virus injection in mice. Invest Ophthalmol Vis Sci.

[67] Mombaerts P (1992). RAG-1-deficient mice have no mature B and T lymphocytes. Cell.

[68] Copland DA (2012). Therapeutic dosing of fingolimod (FTY720) prevents cell infiltration, rapidly suppresses ocular inflammation, and maintains the blood-ocular barrier. Am J Pathol.

[69] Forrester JV, Xu H (2012). Good news-bad news: the yin and yang of immune privilege in the eye. Front Immunol.

[70] McMenamin PG (1997). Resident and infiltrating cells in the rat iris during the early stages of experimental melanin protein-induced uveitis (EMIU). Ocul Immunol Inflamm.

[71] Williamson JS (1989). Immunoregulatory properties of bone marrow-derived cells in the iris and ciliary body. Immunology.

[72] Masopust D, Soerens AG (2019). Tissue-resident T cells and other resident leukocytes. Annu Rev Immunol.

[73] Szabo PA (2019). Location, location, location: Tissue resident memory T cells in mice and humans. Sci Immunol.

[74] St Leger AJ (2017). An ocular commensal protects against corneal infection by driving an interleukin-17 response from mucosal γδ T cells. Immunity.

[75] Zhao Z (2017). Choroidal γδ T cells in protection against retinal pigment epithelium and retinal injury. FASEB J.

[76] Parker ME, Ciofani M (2020). Regulation of γδ T cell effector diversification in the thymus. Front Immunol.

[77] Haas JD (2012). Development of interleukin-17-producing γδ T cells is restricted to a functional embryonic wave. Immunity.

[78] Vantourout P, Hayday A (2013). Six-of-the-best: unique contributions of γδ T cells to immunology. Nat Rev Immunol.

[79] Ribot JC (2021). γδ T cells in tissue physiology and surveillance. Nat Rev Immunol.

[80] Ribeiro M (2019). Meningeal γδ T cell-derived IL-17 controls synaptic plasticity and short-term memory. Sci Immunol.

[81] Chen HC (2019). IL-7-dependent compositional changes within the γδ T cell pool in lymph nodes during ageing lead to an unbalanced anti-tumour response. EMBO Rep.

[82] Kallemeijn MJ (2018). Next-generation sequencing analysis of the human TCRγδ^+^ T-cell repertoire reveals shifts in Vγ- and Vδ-usage in memory populations upon aging. Front Immunol.

[83] Chen L (2020). Skin expression of IL-23 drives the development of psoriasis and psoriatic arthritis in mice. Sci Rep.

[84] Weigelt CM (2021). AAV-mediated expression of human VEGF, TNF-α, and IL-6 induces retinal pathology in mice. Transl Vis Sci Technol.

[85] Weigelt CM (2022). Characterization and validation of in vitro and in vivo models to investigate TNF-α-induced inflammation in retinal diseases. Transl Vis Sci Technol.

[86] Xu J (2020). Aqueous cytokine levels in four common uveitis entities. Int Immunopharmacol.

[87] Sandrock I (2018). Genetic models reveal origin, persistence and non-redundant functions of IL-17–producing γδ T cells. J Exp Med.

[88] Munoz LD (2020). Skin resident γδ T cell function and regulation in wound repair. Int J Mol Sci.

[89] Papotto PH (2017). IL-23 drives differentiation of peripheral γδ17 T cells from adult bone marrow-derived precursors. EMBO Rep.

[91] Cosmi L (2008). Human interleukin 17-producing cells originate from a CD161^+^CD4^+^ T cell precursor. J Exp Med.

[92] Lou B (2022). A single-cell transcriptomic atlas of the human ciliary body. Cell Mol Life Sci.

[93] van Zyl T (2022). Cell atlas of the human ocular anterior segment: Tissue-specific and shared cell types. Proc Natl Acad Sci U S A.

[94] Hassman L (2023). Single cell RNA-Sequencing of aqueous immune cells reveals a spectrum of ocular immune responses in human uveitis. Invest Ophthalmol Vis Sci.

[95] Cua DJ, Tato CM (2010). Innate IL-17–producing cells: the sentinels of the immune system. Nat Rev Immunol.

[96] Pepple KL, Lin P (2018). Targeting interleukin-23 in the treatment of noninfectious uveitis. Ophthalmology.

[97] Arepalli S, Rosenbaum JT (2019). The use of biologics for uveitis associated with spondyloarthritis. Curr Opin Rheumatol.

[98] Mattapallil MJ (2012). The Rd8 mutation of the Crb1 gene is present in vendor lines of C57BL/6N mice and embryonic stem cells, and confounds ocular induced mutant phenotypes. Invest Ophthalmol Vis Sci.

[99] Chu CJ (2016). Multimodal analysis of ocular inflammation using the endotoxin-induced uveitis mouse model. Dis Model Mech.

[100] Kerr EC (2008). Analysis of retinal cellular infiltrate in experimental autoimmune uveoretinitis reveals multiple regulatory cell populations. J Autoimmun.

