# Supplementary material for: IL-23 drives uveitis by acting on a population of tissue-resident entheseal T cells
Source: JCI Insight. 2025 Aug 28;10(19):e182616. doi: 10.1172/jci.insight.182616 (PMC12513492; doi:10.1172/jci.insight.182616)
Supplement: Supplemental data [file jciinsight-10-182616-s207.pdf]

Supplemental Acknowledgements:

Names and affiliations of additional members of the ORBIT Consortium not listed as co-authors

ORBIT — The Kennedy Institute of Rheumatology

Dr. Andrew Foers; Program Manager and postdoctoral fellow, Kennedy Institute of Rheumatology
Professor Alastair Denniston; Consultant ophthalmologist (eye specialist) at University Hospitals Birmingham
Dr. Srilakshmi Sharma; MRC fellow at the Kennedy Institute and Consultant Ophthalmologist at Oxford University Hospitals NHS Trust.
Dr. Ian Reekie; Clinical Research Fellow in Rheumatology, at the Kennedy Institute of Rheumatology
Dr. Lakshanie Wickramasinghe; Postdoctoral Fellow, Kennedy Institute of Rheumatology

Supplemental Methods

Synthesis of mouse 'hyper-IL-23' cytokine sequence

The sequence synthesis was performed by GenScript, Piscataway, USA. Mouse IL-23 artificial hyperkine (a term coined by the DNAX Research institute that describes two protein subunits connected by a synthetic linker - GSGSSRGGSGSGGSGGGGSKL), consisting of mouse IL-12p40 and p19 as one sequence (1), with flanking restriction enzyme sites and ligated into a pD10-CMV plasmid **(Supplementary Figure 3A)**. In animal models, this 'hyper-IL-23' cytokine sequence has been shown to drive pathology strongly resembling human autoimmune inflammatory conditions including psoriasis and AS (2, 3). pCMV-eGFP, was also used in this study, and was a gift from Connie Cepko (Addgene plasmid #11153; http://n2t.net/addgene:11153; RRID: Addgene_11153).

AAV Production

AAV vectors were either purchased from Vector Biolabs (PA, USA) or manufactured at the University of Bristol as previously described (4). In brief, recombinant ShH10 serotype particles were produced through triple-plasmid transfection using PEI transfection reagent into 293T-HEK cells. ShH10 particles were bound to a 1-mL HiTrap AVB Sepharose column (GE Healthcare, USA) and eluted with 50 mM glycine (pH 2.7) into 1 M Tris (pH 8.8). Vectors were desalted and concentrated in PBS-MK to a concentration of 1 x 10^{13} genome copies per milliliter (gc/mL) using a Vivaspin 4 (10 kDa) concentrator. Vector genome titers were determined by quantitative real-time PCR using probes binding to ITR sequences **(Supplementary Table 1)**. An amplicon-based standard series of known concentration was used for sample interpolation. Preparations were certified as endotoxin <5 EU/mL by Pyrotell-T kinetic turbidimetric endotoxin test (Associates of Cape Cod, MA, USA). Vectors used in the study were termed ShH10_IL-23 (expressing 'hyper-IL-23' cytokine) or ShH10_EGFP (control vector expressing GFP).

ELISA

A mouse IL-23 Quantikine ELISA kit (Biotechne, USA) was used to quantify levels of IL-23 according to manufacturer's instructions. Homogenised ocular samples were spun at 13,000 rpm for 10 minutes and the supernatant typically diluted between 1:10 to 1:1000 prior to testing. Samples were analysed with technical triplicates unless limited material precluded this. This assay has a detection range of 15.6 - 1,000 pg/mL, recognizing natural and recombinant mouse IL-23, but not free p19 or p40 subunits.

Histology

Murine ocular tissues

***Immunofluorescence imaging on tissue sections*:** For immunofluorescent imaging, mouse eyes (B6(Cg)-Tyrc-2J/J (Albino), C57BL/6J or IL-23R-eGFP(+/-) were fixed with 4% paraformaldehyde, frozen in optical cutting temperature compound (VWR, PA, USA), and sectioned at 12-μm intervals. Slides were incubated with a 1:1,000 dilution of DAPI (Sigma

Aldrich, UK) and mounted in fluorescence mounting media (Agilent Technologies, CA, USA) before imaging on an EVOS FL microscope (Thermo Fisher Scientific, UK) or Leica SP5 Confocal microscope (Leica Microsystems, Germany).

Whole tissue anterior segment Lightsheet imaging: Following euthanasia, 8-week-old *B6(Cg)-Tyrc-2J/J* mice underwent cardiac perfusion with 4% Paraformaldehyde. Eyes were enucleated and the anterior segment isolated by dissection along the ocular equator. The anterior segments were then placed overnight in 1:4 dilution of BD Cytofix/Cytoperm in PBS at 4°C. The following day this was washed once in PBS then incubated for 24 hours at room temperature in 1x BD Wash Perm buffer with 5% normal goat Serum (Jackson Immunolabs). This was then replaced with 1x BD Wash Perm buffer, with diluted AlexaFluor 647 conjugated rat anti-mouse CD3 antibody (1:100; 101010; Clone [17A2]) and incubated for 3 days at room temperature. The tissue was washed for 4 hours then placed into Ce3D clearing medium (5) overnight, before mounting in Ce3D medium and imaging on a Z1 Lightsheet system (Zeiss) acquiring the pre-set 633nm and 488nm channels. Images underwent 3D reconstruction using Imaris version 8.0 (Oxford Instruments).

Human ocular tissues

Human ocular tissues were fixed in 10% buffered formalin, before paraffin embedding, sectioning at 4 μm thickness onto adhesive glass slides (Leica, # 3800050) and baked at 60°C for 30 mins and then at 37°C for a further 60 mins.

Antigen retrieval: Slides were incubated at 60°C for 60 min, and tissue sections subjected to deparaffinization and target retrieval steps (heat-mediated antigen retrieval at pH 6) using an automated PT Link instrument (Dako). Antibody staining was performed using the EnVision FLEX visualization system with an Autostainer Link 48 (Dako). Antibody binding was visualized using FLEX 3,3'-diaminobenzidine (DAB) substrate working solution and haematoxylin counterstain (Dako, UK). Primary antibodies against human CD3 (1:100; #A045229-2) and CD68 (1:400; #M0876) were obtained from Dako, UK. Images were acquired using an inverted microscope (Axiovision software -Zeiss). Due to extremely small sample size of the specimens, only four images were acquired: two at x20 and two at x40 magnification.

Immunofluorescence imaging on tissue sections This protocol was adapted from a previous paper (6). Briefly, following antigen retrieval, tissues were blocked in 5% normal goat serum (Sigma-Aldrich) in PBS for 60 min in a humid chamber at room temperature. After removal of blocking solution, sections were incubated with the primary antibody cocktail diluted in 5% normal goat serum in PBS for 2 hours at room temperature. Details of primary antibodies used for immunofluorescence are listed in **(Supplementary Table 6)**. Sections were washed (3x PBS–Tween 20 (PBST) for 5 min), the incubated with secondary antibodies each diluted 1:200 in 5% normal equine serum (Sigma-Aldrich) in PBS for 2 hours. The secondary antibodies were Alexa Fluor goat anti-mouse IgG2a or IgG2b or goat anti-rabbit IgG (Life Technologies) and goat anti-

mouse IgG1 (Southern Biotech). Sections counterstained with 2 μM POPO-1 nuclear counterstain (Life Technologies), diluted in PBS containing 0.05% saponin (Sigma-Aldrich) for 20 min. Tissue autofluorescence was quenched with a solution of 0.1% Sudan Black B (Applichem) in 70% ethanol for 10 min. Slides were mounted using fluorescent mounting medium (VectaShield). For negative controls, the primary antibody was substituted for universal isotype control antibodies: cocktail of mouse IgG1, IgG2a, IgG2b, IgG3, and IgM (Dako) and rabbit immunoglobulin fraction of serum from non-immunized rabbits (Dako).

Second Harmonic Imaging

Multi-photon images were acquired on a Zeiss LSM 710 laser-scanning confocal coupled to an inverted Zeiss Axio Observer.Z1 microscope. A 40x/1.3 NA oil immersion objective was used with immersion media RI: 1.518. Two-photon excitation was performed using a Chameleon Vision II, Ti:Sapphire laser (Coherent, 680 - 1080 nm, pulse width: 140 fs at peak, repetition rate: 80MHz). Second Harmonic Generation (SHG) imaging was performed by setting the two-photon at 760 nm to reach an excitation wavelength of 380 nm in the sample. Images were acquired with non-descanned detectors (NDD) using a filter set equipped with a long pass dichroic LP 445 nm and a bandpass filter (BP380-430).

References:

1. Oppmann B, Lesley R, Blom B, Timans JC, Xu Y, Hunte B, et al. Novel p19 protein engages IL-12p40 to form a cytokine, IL-23, with biological activities similar as well as distinct from IL-12. *Immunity*. 2000;13(5):715-25.
2. Sherlock JP, Joyce-Shaikh B, Turner SP, Chao CC, Sathe M, Grein J, et al. IL-23 induces spondyloarthropathy by acting on ROR-gammat+ CD3+CD4-CD8- entheseal resident T cells. *Nat Med*. 2012;18(7):1069-76.
3. Chan JR, Blumenschein W, Murphy E, Diveu C, Wiekowski M, Abbondanzo S, et al. IL-23 stimulates epidermal hyperplasia via TNF and IL-20R2-dependent mechanisms with implications for psoriasis pathogenesis. *J Exp Med*. 2006;203(12):2577-87.
4. Gonzalez-Cordero A, Goh D, Kruczek K, Naeem A, Fernando M, Kleine Holthaus SM, et al. Assessment of AAV Vector Tropisms for Mouse and Human Pluripotent Stem Cell-Derived RPE and Photoreceptor Cells. *Hum Gene Ther*. 2018;29(10):1124-39.
5. Li W, Germain RN, and Gerner MY. High-dimensional cell-level analysis of tissues with Ce3D multiplex volume imaging. *Nat Protoc*. 2019;14(6):1708-33.
6. Dakin SG, Buckley CD, Al-Mossawi MH, Hedley R, Martinez FO, Wheway K, et al. Persistent stromal fibroblast activation is present in chronic tendinopathy. *Arthritis Res Ther*. 2017;19(1):16.

IL-23 drives uveitis by acting on a novel population of tissue resident entheseal T cells

Robert Hedley*[1,2], Amy Ward*[3], Colin J Chu[3,4] Sarah E Coupland[5], Serafim Kiriakidis[1], Peter C Taylor[1], Stephanie G Dakin[1], The ORBIT consortium[6], Christopher D Buckley[7], Jonathan Sherlock[7,8], Andrew D Dick[$ 3,4] & David A Copland[$ 3,4]

Supplementary Figures:

Supplementary Figure 1*: CD3+ cells located in tissues of the naïve mouse anterior uvea

Supplementary Figure 2: Phenotyping of CD3+ γδTCR+IL-23R+ T cells in the naïve eye

Supplementary Figure 3: ShH10 IL-23 AAV production and purification process

Supplementary Figure 4: Infiltrating CD45+ cells comprise both adaptive and innate immune cell types

Supplementary Figure 5: Extended time-course demonstrates intravitreal delivery ShH10_IL-23 AAV leads to chronic, persistent inflammation at Day 50

Supplementary Figure 6: αβ T cells including IL-17A+ and IFN-γ subsets are recruited to the anterior compartment in response to IL-23-mediated activation of resident γδ T cells

Supplementary Figure 7: γδ T cells remain as the primary IL-23R+IL-17A+ in response to IL-23 exposure

Supplementary Figure 8: Tissue resident CD3+ T cells present in limbal sclera and ciliary body

Supplementary Figure 9: The healthy human limbal sclera is home to a diverse array of CD45+ leukocytes

Supplementary Figure 1: CD3+ cells located in tissues of the naïve mouse anterior uvea.
Naïve B6(Cg)-Tyrc-2J/J (albino) mice were perfused, and intact globes optically cleared, immunolabelled with CD3e and DAPI. Lightsheet Z.1 acquired immunofluorescent whole mount images captured as movie format 3D rendered video shows expression of tissue autofluorescence (white) and CD3+ T cells (purple). Co-expression of CD3 and nuclei can be seen within different regions including the peripheral cornea, limbal sclera and ciliary body. Images captured at x10 magnification.

***MP4 file has been uploaded separately as video**

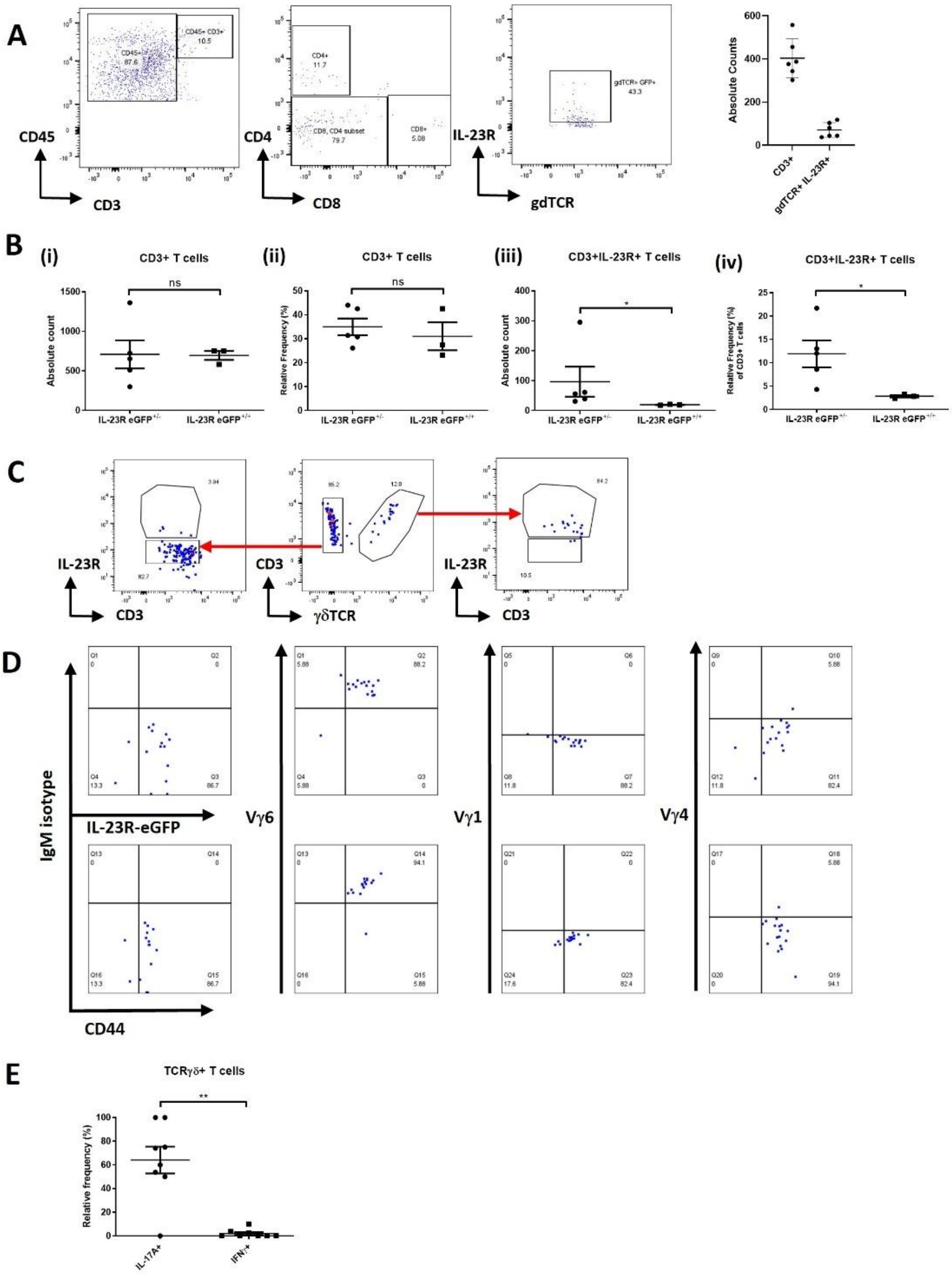

Supplementary Figure 2: Phenotyping of CD3+ $\gamma\delta$TCR+IL-23R+ T cells in the naïve eye.

(A) B6(Cg)-Tyrc-2J/J xIL-23Re GFP+/- (Albino background) show similar CD3+ and $\gamma\delta$TCR+IL-23R+ absolute counts in naïve anterior uvea (AU) (n=6). **(B)** Functional IL-23R expression may represent an important determinant in the accumulation and/or residency of ocular CD3+ $\gamma\delta$TCR+IL-23R+ T cells. FACS compiled frequency data of pooled left and right AU tissue collected from IL-23R eGFP+/- (heterozygous) or IL-23R eGFP+/+ (homozygous) mice (n = 5). The absolute count and relative frequency of viable CD3+ T cells ((i) & (ii)) and CD3+$\gamma\delta$TCR+IL-23R+ cells. Line represents mean and error bars show SEM. * = *P*<0.05 (unpaired Student's two-tailed t-test). **(C)** Representative flow cytometry gating to demonstrate IL-23R expression is restricted to $\gamma\delta$TCR+ and not $\alpha\beta$TCR+ resident cells in naïve AU. **(D)** Frequencies of anterior CD3+$\gamma\delta$TCR+ expressing Vγ1, Vγ4 or Vγ6, as determined by FACS analysis on gated IL-23R+ or CD44+ cells isolated from the naïve AU of IL-23ReGFP+/- reporter mice. Dot plots depict pooled data (two eyes from single mouse), representative of 2 independent experiments. **(E)** FACS and intracellular cytokine staining of CD3+$\gamma\delta$TCR+ cells from AU samples at Day 12 post-injection of ShH10 IL-23 (1E11vg/eye). Cells were stimulated with PMA/ionomycin and IL-17A and IFN-g production shown in compiled frequency plots symbols represent pooled left and right eyes from individual mice. Data shown as means ± SEM and are representative of single experiment. ns=not significant, $*$ = P < 0.05 $**$ = P < 0.01, unpaired t-test.

.

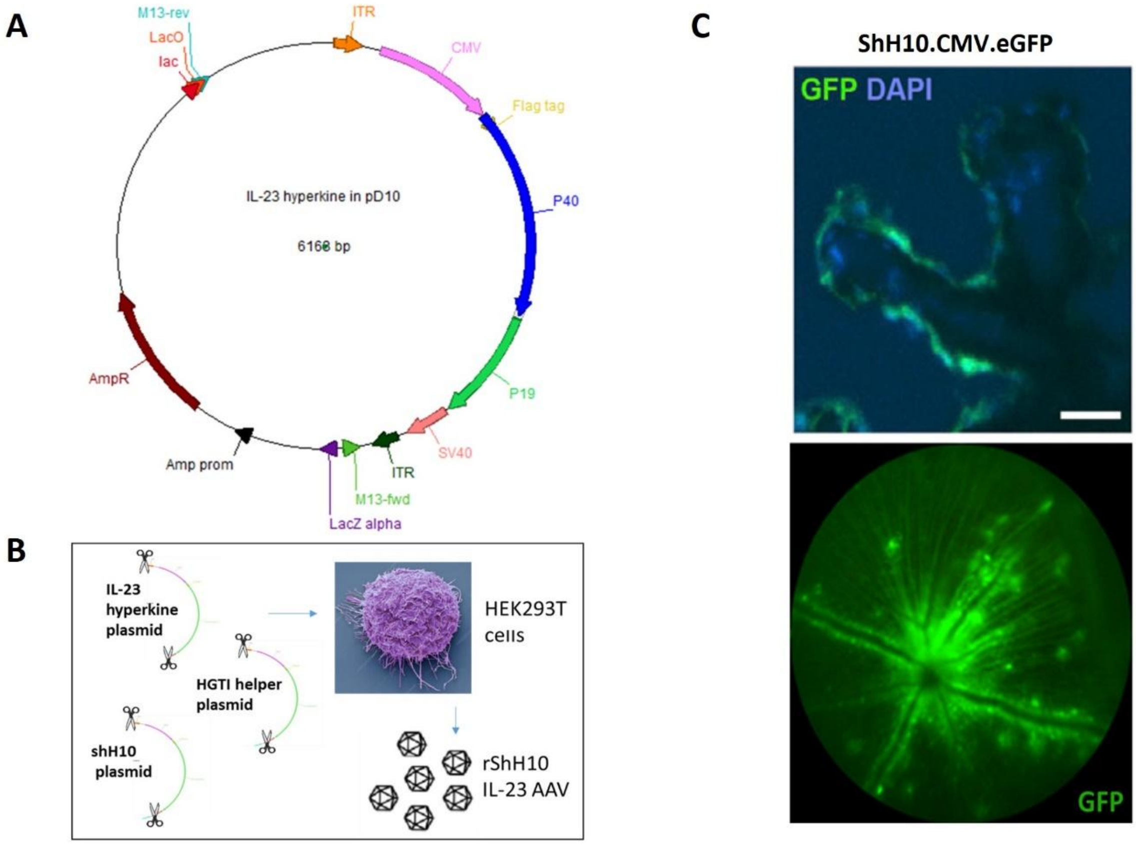

Supplementary Figure 3: ShH10 IL-23 AAV production and purification process.

Generation of adeno-associated virus (AAV) ShH10 serotype encoding ‘hyper-IL-23’ cytokine transgene (ShH10 IL-23 AAV). **(A)** Schematic diagram of mouse IL-23 sequence (consisting of p40 and p19 subunits [60]) ligated into a pD10-CMV plasmid. **(B)** HEK-293T cells seeded at 4.5x10^{6} in Dublecco’s modified eagle medium (DMEM) with 10% Performance Plus Fetal Bovine Serum and 1x Antibiotic-Antimycotic (Invitrogen, life technologies, Paisley, UK) were simultaneously transfected with 10µg of pD10 genomic plasmid, 10µg of ShH10 capsid plasmid and 30µg of pHGTI helper plasmid per plate using Polyethylenimine (PEI). The cells were harvested after 72 hours and purified for viral ShH10 vector. **(C)** The ShH10 capsid serotype permits rapid transduction of the ciliary body, and cells of the inner retina when injected into the mouse eye via the intravitreal route. Section of ciliary body (Wu, J., et al., *Gene Therapy for Glaucoma by Ciliary Body Aquaporin 1 Disruption Using CRISPR-Cas9*. Mol Ther, 2020. **28**(3): p. 820-829), and retinal fundus image showing ShH10.CMV.eGFP transduction at day 12 post-injection.

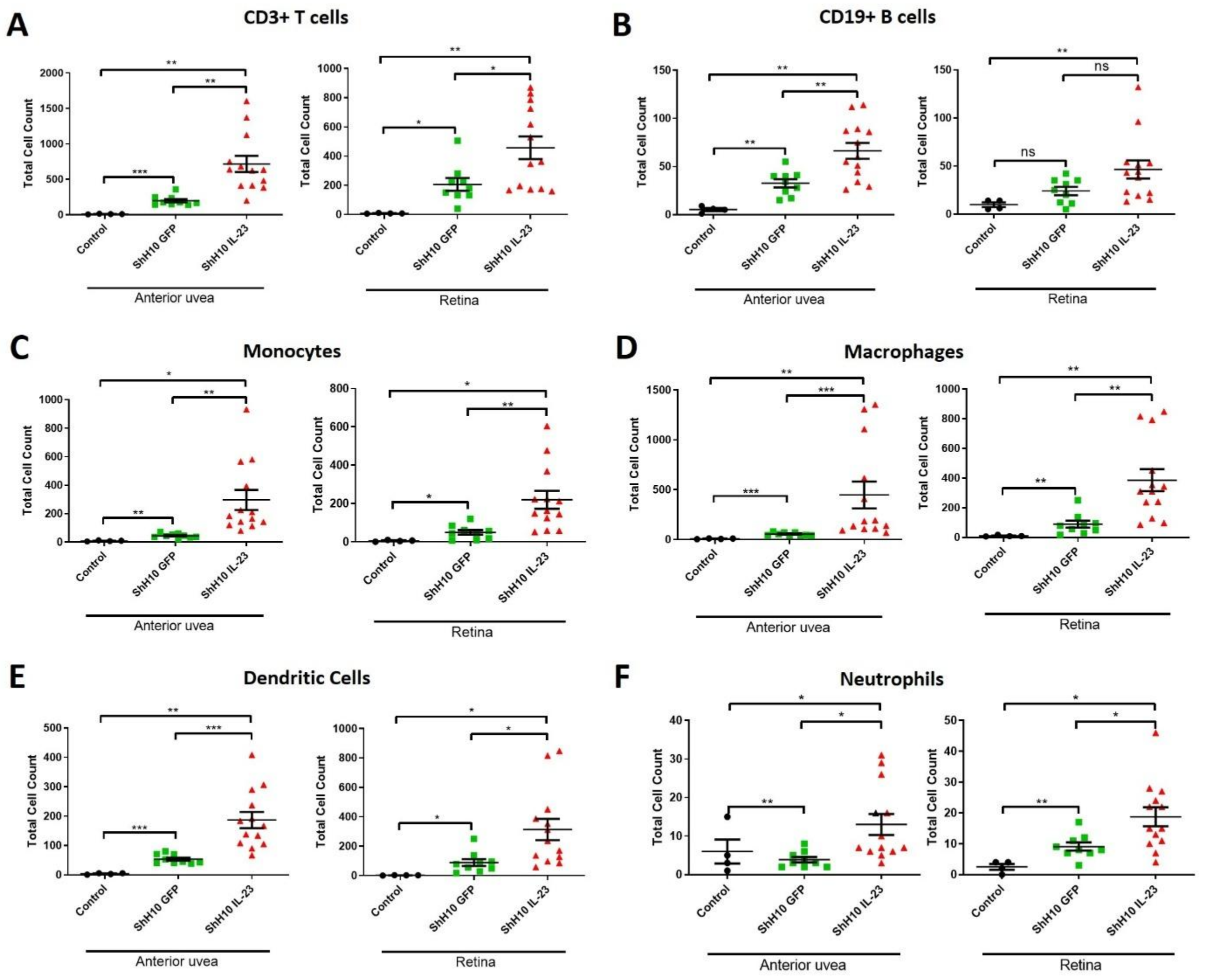

Supplementary Figure 4: Infiltrating CD45+ cells comprise both adaptive and innate immune cell types. Flow cytometric analysis of anterior uvea and retina samples collected at day 12 post intravitreal injection of ShH10.GFP or ShH10.IL-23 [1E11vg/eye]. CD45+ infiltrate comprises both adaptive and innate immune cell populations including **(A)** CD3+ T cells, **(B)** CD19+ B cells, **(C)** CD11b+F4/80- Monocytes, **(D)** CD11b+F4/80+ Macrophages, **(E)** CD11c+F4/80- Dendritic cells, and **(F)** Ly6G+ Neutrophils. Mean count is represented by the central line with error bars showing SEM. * = P<0.05, ** = P<0.01, and *** = P<0.001 (unpaired Student's two-tailed t-test).

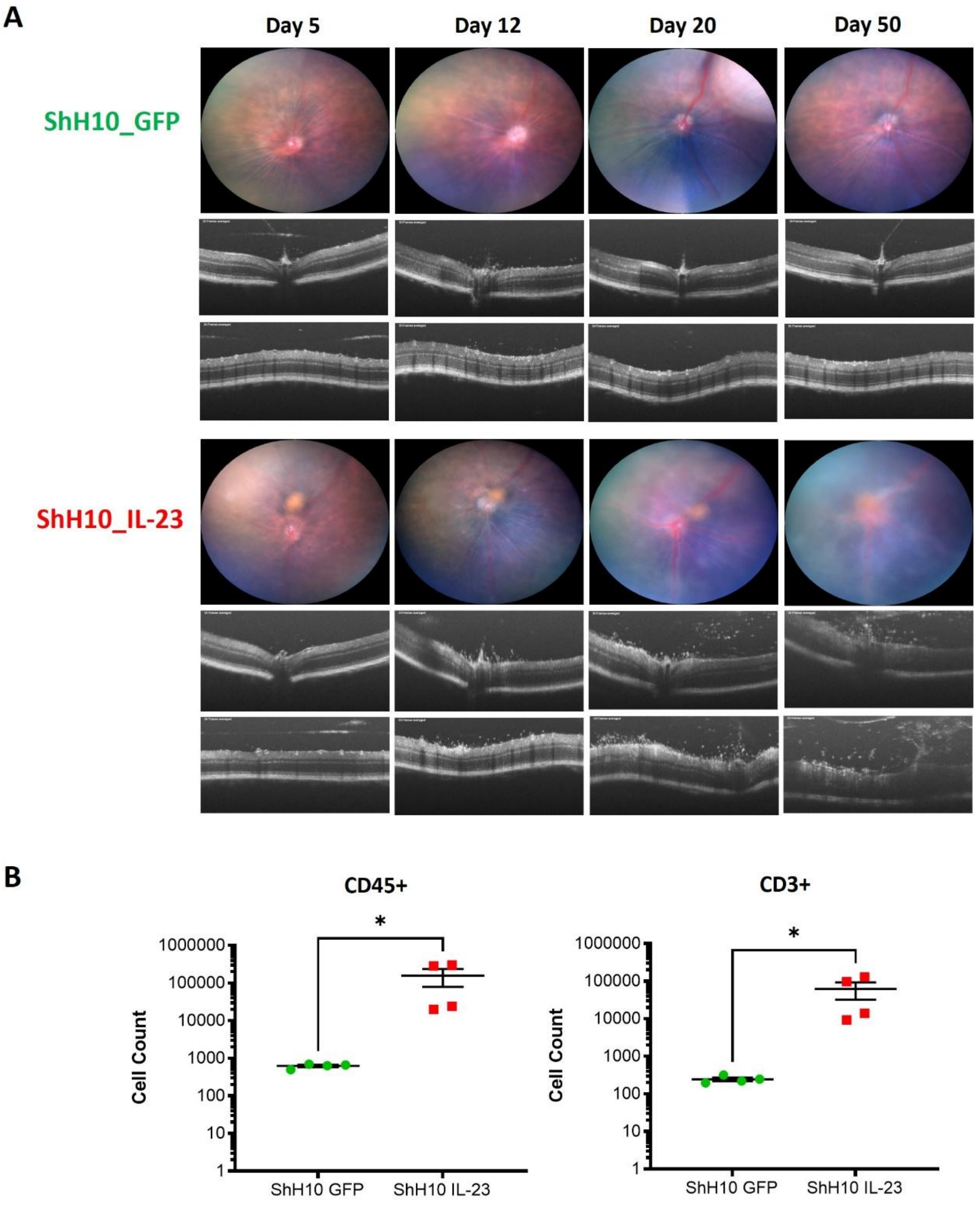

Supplementary Figure 5: Extended time-course demonstrates intravitreal delivery ShH10_IL-23 AAV leads to chronic, persistent inflammation at Day 50.

Wild-type C57BL/6J mice (n=4) received intravitreal injection of $1x10^{11}$ vector genomes of ShH10_IL23, with ShH10_GFP (control AAV) administered to the contralateral eye. **(A)** Mice were clinically monitored (Fundus and OCT) and representative images from single mouse are shown for days 5, 12, 20 & 50 post-injection. On day 50, enucleated eyes were dissected and prepared for flow cytometric immune phenotyping. **(B)** Graphs show total live counts for CD45+ and CD3 cells from combined anterior uvea and retina obtained from individual eyes. Statistical analysis: Mann Whitney test; *p<0.05; Data shown as mean ± SEMs and representative of a single experiment.

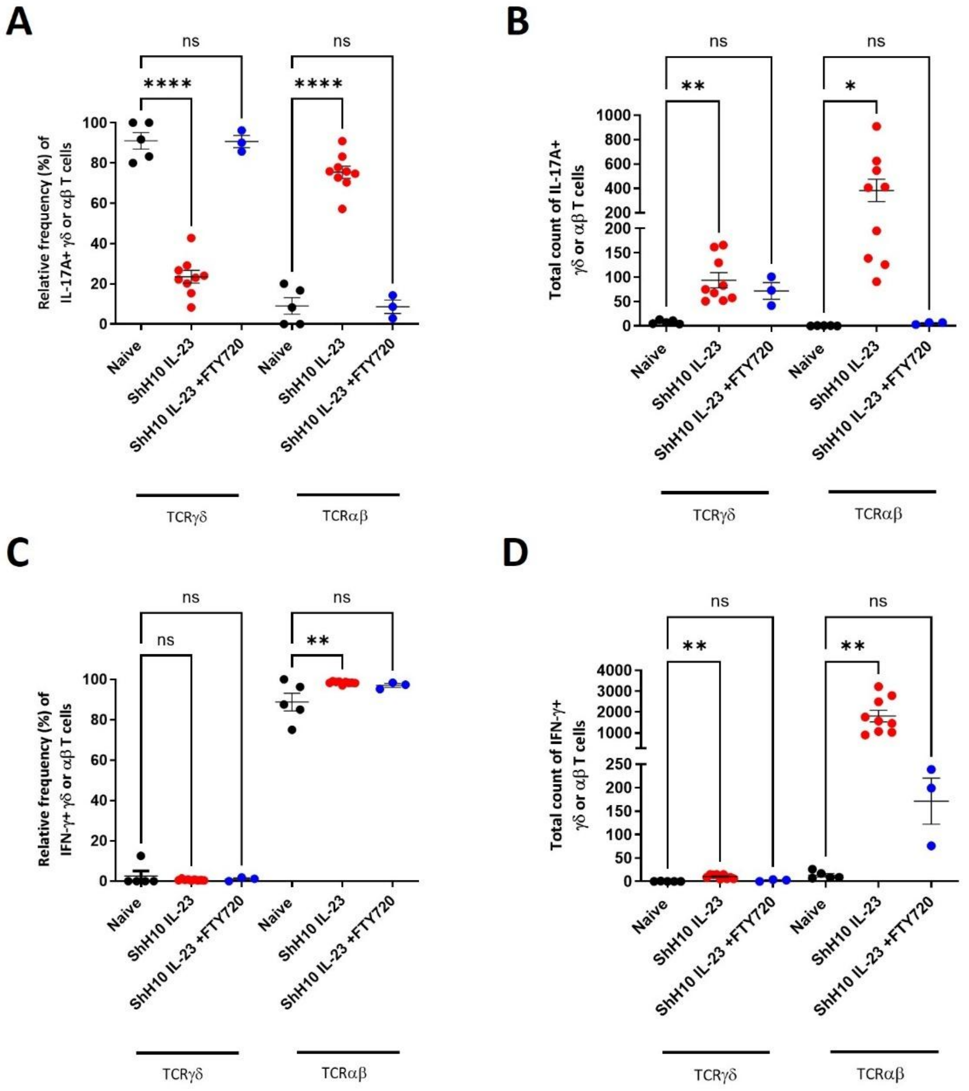

Supplementary Figure 6: αβ T cells including IL-17+ and IFN-γ subsets are recruited to the anterior compartment in response to IL-23-mediated activation of resident γδ T cells

Flow cytometric ICCS phenotyping of γδ and αβ T cell subsets in the anterior uvea. IL-23R-eGFP(+/-) eyes from naïve controls (un-injected) or mice injected with ShH10_IL23 ($1x10^{11}$ vector genomes) receiving oral dosing with Fingolimod (FTY720; 10mg/kg) or vehicle control, administered on alternate days following AAV injection. Following dissection on day 12, ex vivo AU samples were stimulated (PMA/ionomycin) and stained for surface markers (CD45, CD3, γδ TCR,) and intracellular cytokines (IL-17A and IFN-γ) **(A, B)** Relative frequency and total counts for IL-17A+ γδ and αβ subsets. **(C, D)** Relative frequency and total counts for IFN-γ+ γδ and αβ subsets. Statistical analysis; One-way ANOVA; Data expressed as means +/- SEM; ns = not significant, ** = *P*<0.01, *** = *P*<0.001, **** *P*<0.0001.

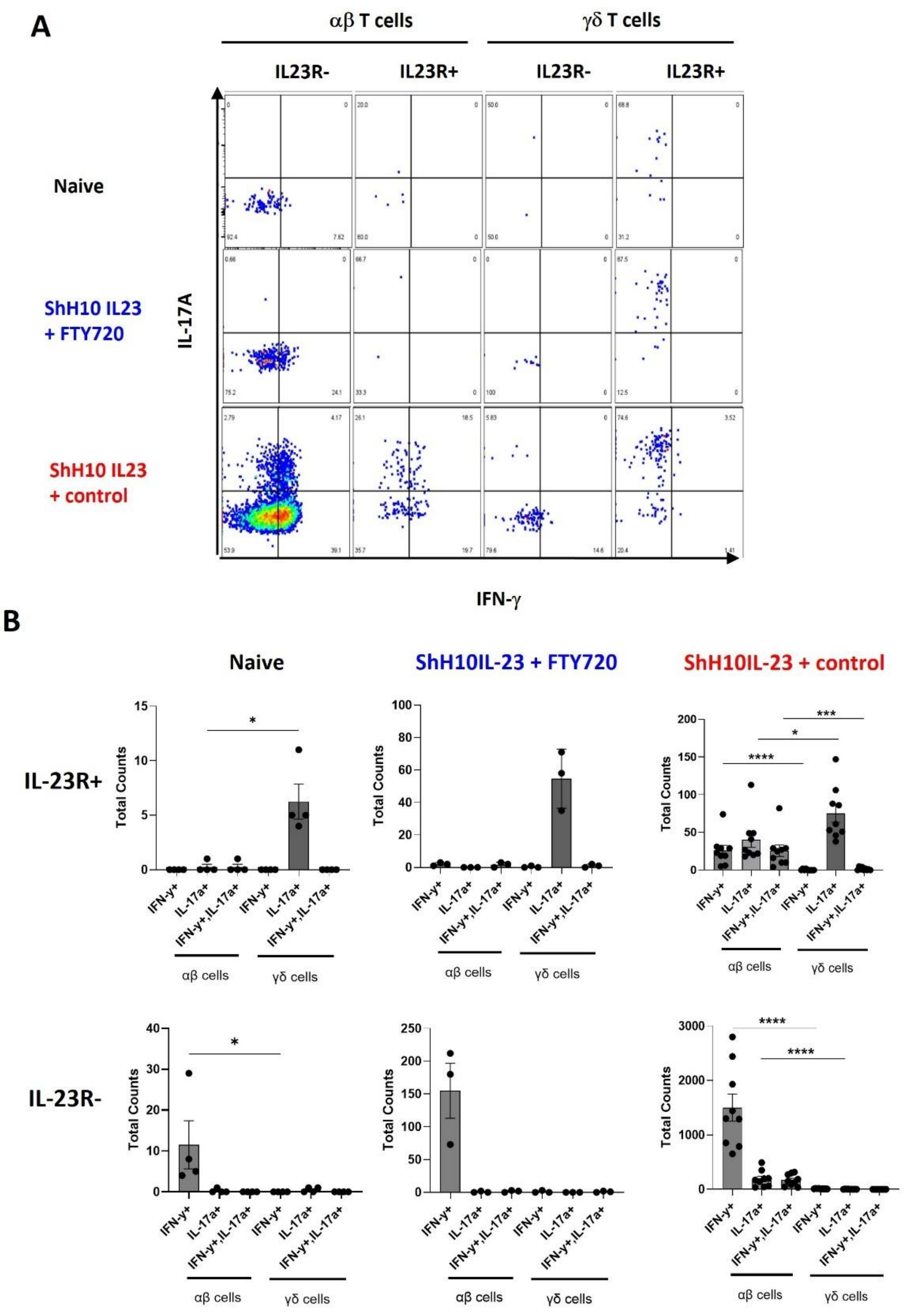

Supplementary Figure 7: γδ T cells remain as the primary IL-23R+IL-17A+ in response to IL-23 exposure
Flow cytometric ICCS phenotyping of αβ and γδ T cell subsets in the anterior uvea. IL-23R-eGFP(+/-) eyes from naïve controls (un-injected) or mice injected with ShH10_IL23 ($1x10^{11}$ vector genomes) receiving oral dosing with Fingolimod (FTY720; 10mg/kg) or vehicle control, administered on alternate days following AAV injection. Following dissection on day 12, ex vivo AU samples were stimulated (PMA/ionomycin) and stained for surface markers (CD45, CD3, γδ TCR,) and intracellular cytokines (IL-17A and IFN-γ). Cells were gated based on IL-23R- vs IL-23R+ expression on αβ and γδ T cell subsets. **(A)** Representative flow cytometry plots to show cytokine profile and **(B)** total cell counts of IFN-γ+, IL-17A+ or IFN-γ/IL-17A double-positive cells. Mann Whitney test; *p<0.05 ***p<0.001, ****p<0.0001; Data shown as mean ± SEMs and representative of a single experiment.

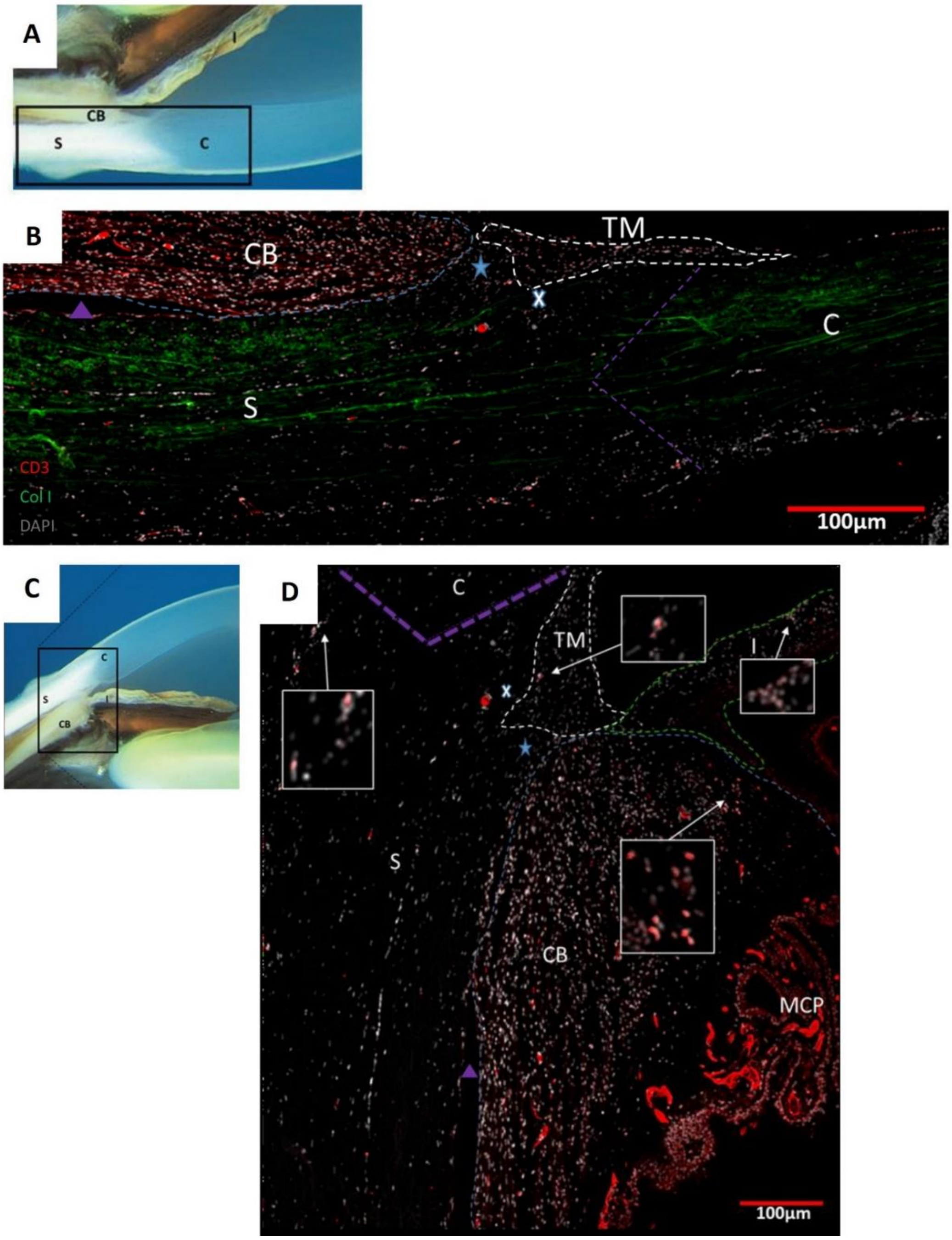

Supplementary Figure 8: Tissue resident CD3+ T cells present in limbal sclera and ciliary body

FFPE tissue sections from post-mortem human eyes were obtained from the Liverpool eye bank for immunostaining. **(A, C)** Photographic images to show the tissue location and orientation of the corresponding immunofluorescence image [94]. C – cornea, S – sclera, CB- ciliary body. **(B)** Immunofluorescent staining shows nucleus (grey), collagen type 1 (Col1; green), and CD3+ cells (red). **(D)** Immunofluorescent staining for nucleus (grey) and CD3+ (red) T cells. White arrows and boxes show areas of T cell clusters under digital magnification. Images comprise tile scan composed of 4 x 4 individual images, captured at x10 magnification. C – cornea, S – sclera, purple line – corneoscleral limbus, 'TM' and white line – trabecular meshwork, 'X' – Schlemm's canal, blue star – scleral spur, purple triangle - supraciliary space, 'CB' and blue line – ciliary body, and MCP – major ciliary processes. Red scale bar indicates 100µm.

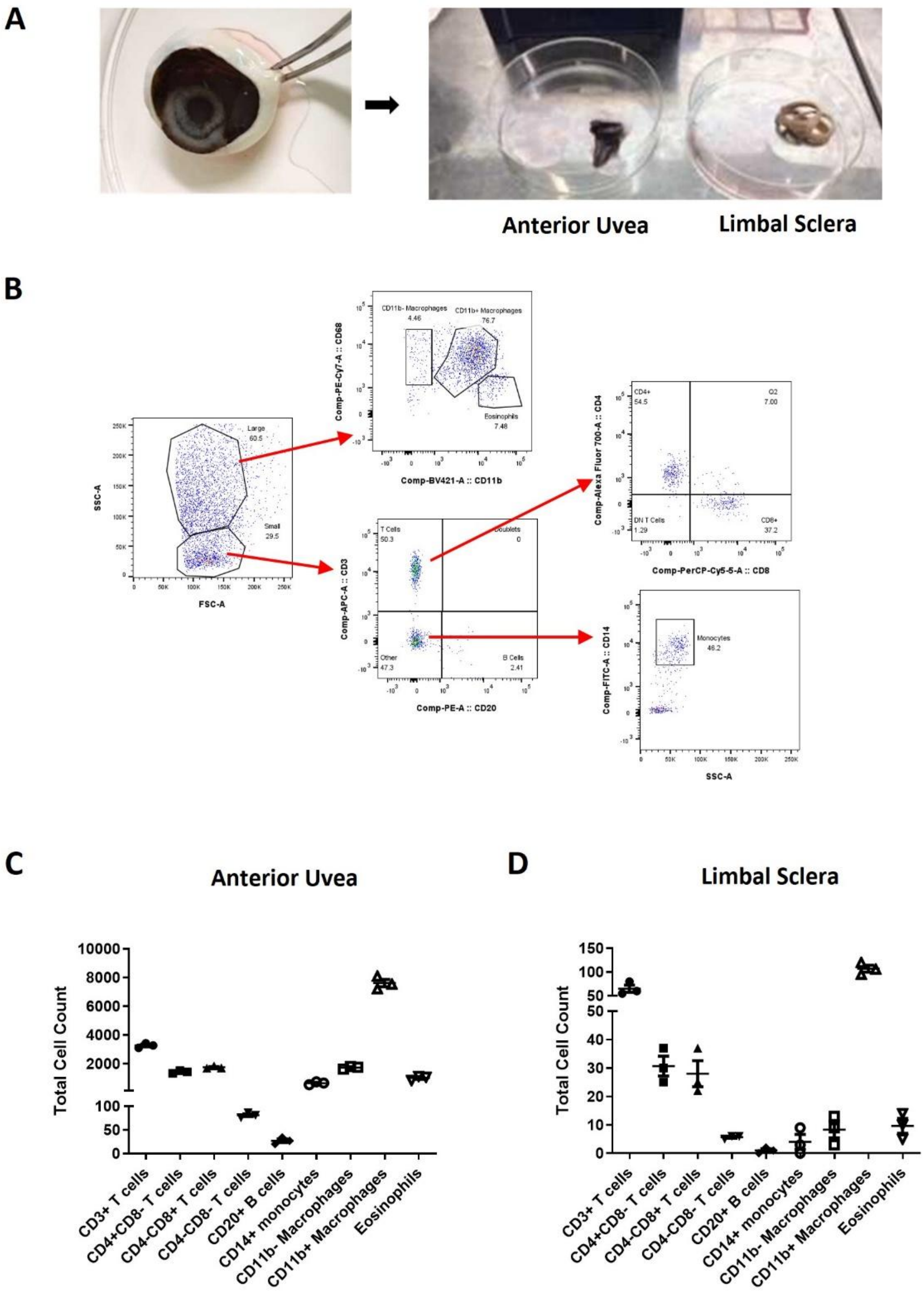

Supplementary Figure 9: The healthy human limbal sclera is home to a diverse array of CD45+ leukocytes. From post-mortem donor eyes, the anterior compartments were dissected to obtain anterior uveal tissue (including iris, ciliary body, trabecular meshwork, and anterior choroid) and limbal sclera (comprising sclera and extra ocular muscle enthesis). Tissues were enzymatically digested, and single cell suspensions generated for FACS immune phenotyping. **(A)** Photographs demonstrating isolation of anterior uvea and limbal sclera from donor eyes with no history of ocular disease. **(B)** Representative plots to show FACS gating strategy for sub populations CD45+ leukocyte populations. **(C, D)** Total counts of CD45+ subsets from AU and LS, which include CD3+ T cells (sub divided into CD4+CD8-,CD4-CD8+,and CD4-CD8- T cells), CD20+ B cells, CD14+ monocytes, CD68+ macrophages (sub divided into CD11b+ and CD11b- Macrophages), and large/high complexity (based on light scatter) CD11b+CD68- cells (Eosinophils).
